# Protons Accumulate at the Graphene–Water Interface

**DOI:** 10.1021/acsnano.5c02053

**Published:** 2025-04-28

**Authors:** Xavier R. Advincula, Kara D. Fong, Angelos Michaelides, Christoph Schran

**Affiliations:** † Yusuf Hamied Department of Chemistry, University of Cambridge, Lensfield Road, Cambridge CB2 1EW, U.K.; ‡ Cavendish Laboratory, Department of Physics, University of Cambridge, Cambridge CB3 0HE, U.K.; § Lennard-Jones Centre, University of Cambridge, Trinity Ln, Cambridge CB2 1TN, U.K.

**Keywords:** protonic defects, nanoconfinement, machine learning potentials, graphene, 2D Materials, molecular simulations

## Abstract

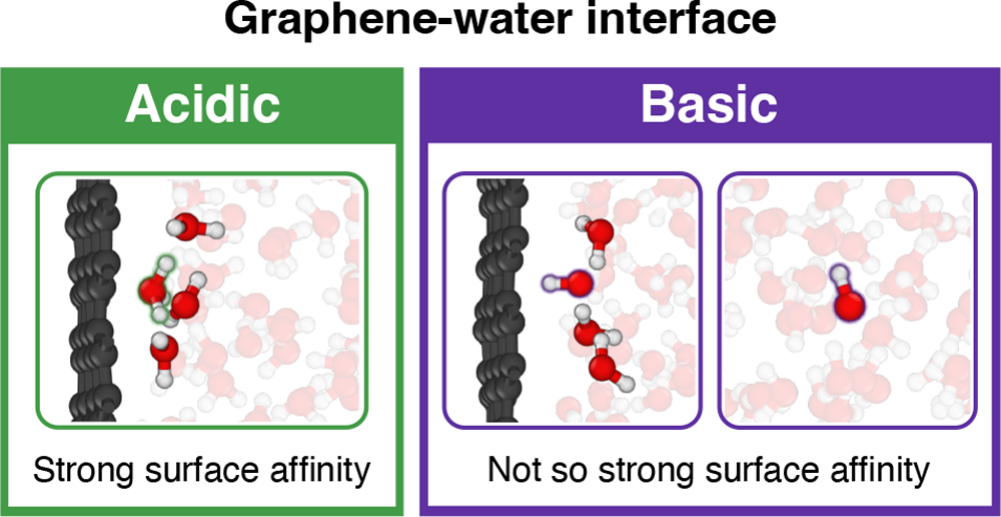

Water’s ability to autoionize into hydroxide and hydronium ions profoundly influences surface properties, rendering interfaces either basic or acidic. While it is well-established that protons show an affinity to the air–water interface, a critical knowledge gap exists in technologically relevant surfaces like the graphene–water interface. Here we use machine learning-based simulations with first-principles accuracy to unravel the behavior of hydroxide and hydronium ions at the graphene–water interface. Our findings reveal that protons accumulate at the graphene–water interface, with the hydronium ion predominantly residing in the first contact layer of water. In contrast, the hydroxide ion exhibits a bimodal distribution, found both near the surface and further away from it. Analysis of the underlying electronic structure reveals local polarization effects, resulting in counterintuitive charge rearrangement. Proton propensity to the graphene–water interface challenges the interpretation of surface experiments and is expected to have far-reaching consequences for ion conductivity, interfacial reactivity, and proton-mediated processes.

Water interacts with interfaces in numerous technologies involving areas such as atmospheric chemistry,^1^ water desalination,^2^ energy production via water splitting,^3^ and storage devices.^4^ For these technologies, understanding the fundamental nature of these interfaces—whether they accumulate or repel ions—is essential to improve performance and facilitate scientific breakthroughs. At the molecular level, this is governed by the self-dissociation of water into hydroxide (OH^–^) and hydronium (H_3_O^+^) ions, which ultimately determines the pH of a solution and facilitates proton transfer. A deeper understanding of how surface interactions influence the propensity for hydronium and hydroxide ions would enable the optimization of these interfacial processes.

Despite significant progress in understanding proton transfer in bulk water and microsolvation,^5−11^ describing these processes near interfaces continue to pose both theoretical and experimental challenges. One of the most enduring and fundamental debates in chemistry has been the nature of the excess proton (hydronium ion) and hydroxide ion at the air–water interface.^12−16^ The main complexity stems from the dynamic nature of protonic defects, namely hydronium and hydroxide ions, in the aqueous phase.^17−19^ Furthermore, the interplay between directional hydrogen bonds and nondirectional van der Waals forces often results in molecular conformations with similar energies,^17−19^ adding to the challenge. Experimental analysis is further complicated by varying probing resolutions and interfacial depths.^14^ Only recently has it been established, both theoretically and experimentally, that the air–water interface accumulates protons, while hydroxide ions are repelled.^20−23^ However, this understanding may not extend to other technologically relevant interfaces, as the fundamental mechanisms governing the behavior of these ions have been reported to differ across various interfaces.^24−29^

Among all interfaces, the graphene–water interface is particularly relevant due to its extensive range of technological applications, from nanofluidics to electronic devices. In recent years, it has been reported that when water is confined between graphene sheets, it exhibits unique properties, including an anomalously low dielectric constant,^30,31^ atypical friction behavior,^32^ and superionic character,^33,34^ among other phenomena.^35,36^ Additionally, graphene’s precise synthesis in experimental settings^37^ makes it an ideal candidate for providing insights applicable to real-world systems. However, our current understanding is limited, and previous studies have shown conflicting results. Grosjean et al.^38^ reported a physisorbed state of hydroxide in the contact layer at the graphene/water interface, which is rationalized in the context of a macroscopic experimental observation^39^ and has significant implications for conductance. Conversely, de Aquino et al.^40^ indicated that hydroxide ions are more prevalent in the interior layers, while hydronium ions are more prevalent in the interfacial layers. More recently, Scalfi et al.^41^ revealed that hydronium adsorbs at the graphene–water interface while hydroxide mostly shows only limited adsorption under specific conditions. While these studies have advanced our understanding, they rely primarily on nonreactive force fields, which may constrain the mechanistic insights they can offer. As a result, despite valuable efforts in the field, a detailed picture of protonic defects near the graphene–water interface has not been established. In particular, detailed mechanistic and thermodynamic insights into hydrogen bonding, the orientational behavior, and interfacial polarization of the hydronium and hydroxide ion at the graphene–water interface have remained unresolved until now. The complex interplay between water and the interface,^42,43^ coupled with the prohibitive computational expense of ab initio molecular dynamics (AIMD) simulations needed to adequately sample these reactive systems, has significantly limited progress in this area. This study aims to address this gap, enhancing our understanding of graphene’s interfacial properties and improving the interpretation of experimental data.

In recent years, machine learning potentials (MLPs) have emerged as an efficient and flexible solution for accurately modeling reactive processes at interfaces. These technologies bypass the prohibitive costs of ab initio calculations, significantly extending the length and time scales accessible in molecular simulations.^44−46^ By accurately representing the potential energy surface of a chosen ab initio reference method, such as density functional theory (DFT), MLPs establish a direct structure–energy relationship. This enables the description of bond-breaking and bond-forming events in complex environments.^47−50^ This approach is particularly beneficial for our study, as classical force field models either lack the reactivity needed to represent the dynamics of covalent OH bond breaking and formation,^51^ or fail to provide the required accuracy, being parametrized mainly for bulk properties.^52^ MLPs address these shortcomings, offering a reliable and accurate method to investigate the specific characteristics of interfacial phenomena.

This work uses MLP-based simulations to demonstrate that protons accumulate at the graphene–water interface, while hydroxide ions exhibit a bimodal distribution, being found both close to the surface and in layers farther from the interface. This surface affinity is due to the hydrogen bond environment of hydronium remaining stable at the interface, while hydroxide’s environment is disrupted. By examining the thermodynamic driving forces, we find that hydronium ions are enthalpically driven to the interface, whereas entropic forces drive hydroxide ions. When comparing these findings to the air–water interface, we see that graphene significantly influences ionic interactions due to polarization effects. This response suggests that macroscopic experiments should be interpreted carefully.

## Results and Discussion

To study protonic defects at the graphene–water interface, we developed an MLP using the MACE architecture.^53^ The MLP demonstrates excellent capability in reproducing the potential energy surface of the underlying DFT at the revPBE-D3^54,55^ level of theory, known to perform well for water^56−58^ and graphene–water^59^ interactions, while also effectively capturing protonic defects.^60^ We have verified the validity of our results with respect to the functional and dispersion correction and confirmed that the observed trends remain unchanged (see Section S2). Our model effectively captures the properties of both types of protonic defects in the water near free-standing graphene surfaces, under conditions ranging from ultraconfined environments to bulk-like settings (see Methods and Section S2 for development and validation details). We used the MLP to perform multinanosecond MD simulations, evaluating properties of five systems involving average slit widths of about 6.5, 9.2, 12.2, 14.7, 19.7 Å. These simulations included one layer (1L), two layers (2L), three layers (3L), four layers (4L), and five layers (5L) of water, each containing either a hydronium or a hydroxide ion (see Section S1).

### Hydronium Resides at the Interface, Hydroxide Does Not

**Figure 1:**
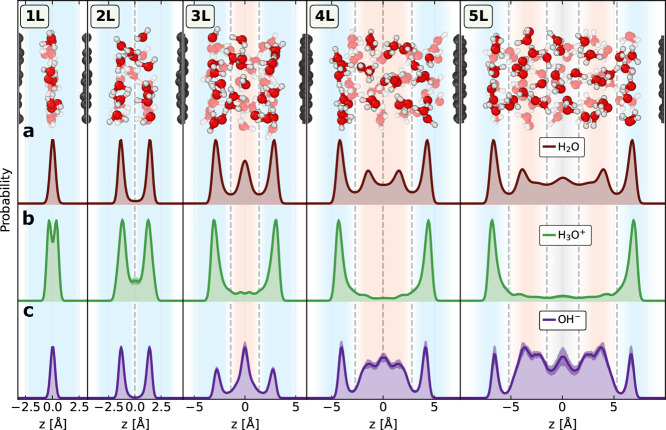
Differences in the surface propensity of hydronium and hydroxide in nanoconfined water. Normalized and symmetrized density profiles along the *z* axis (perpendicular to the free-standing graphene sheets) obtained from the oxygen atoms in neutral water systems (a), and from the specific oxygen of the protonic defect of acidic (b) and basic (c) systems, accompanied by representative snapshots of the neutral water system. For each of the two species and five slit widths, five independent simulations were conducted, each lasting 4 ns. This approach led to a total of 200 ns of cumulative simulation time. The shaded regions indicate the uncertainty resulting from the standard deviation of the five replicate simulations. The background’s faded blue, orange, and gray represent the interfacial, intermediate, and bulk-like water layers, respectively. The vertical dashed lines indicate the partitioning among these water layers. The horizontal axis limits in each plot correspond to the average carbon layer positions.

In Figure 1a, the water density profiles for the 1L–5L systems reveal distinct layers of water near graphene sheets, consistent with observations of water at solid surfaces.^61−65^ Water forms sharply defined ‘interfacial’ layers in direct contact with the graphene sheets across all systems. For the thicker slits, we observe smoother ‘intermediate’ layers, and in the 5L system, these are accompanied by ‘bulk-like’ behavior as the bulk density of water is approached (see Section S2). To investigate the hydronium ion, we analyzed the density profiles from the specific oxygen of the protonic defect. As depicted in Figure 1b, the hydronium ion predominantly resides in the first contact layers of water, the interfacial water layers. Although the hydronium ion is still observed in the water layers further away from the interface, it appears less frequently there. For the hydroxide ion, shown in Figure 1c, the situation is less clear-cut: it can be present either at the interface or in the interior of the film, generally preferring the layers farther from the interface. These patterns are consistent across all studied slit widths featuring an intermediate water region (i.e., 3L–5L). Furthermore, the flexibility of graphene and its impact on these observations have been examined, confirming consistency even with completely rigid graphene sheets as shown in Section S3.

**Figure 2:**
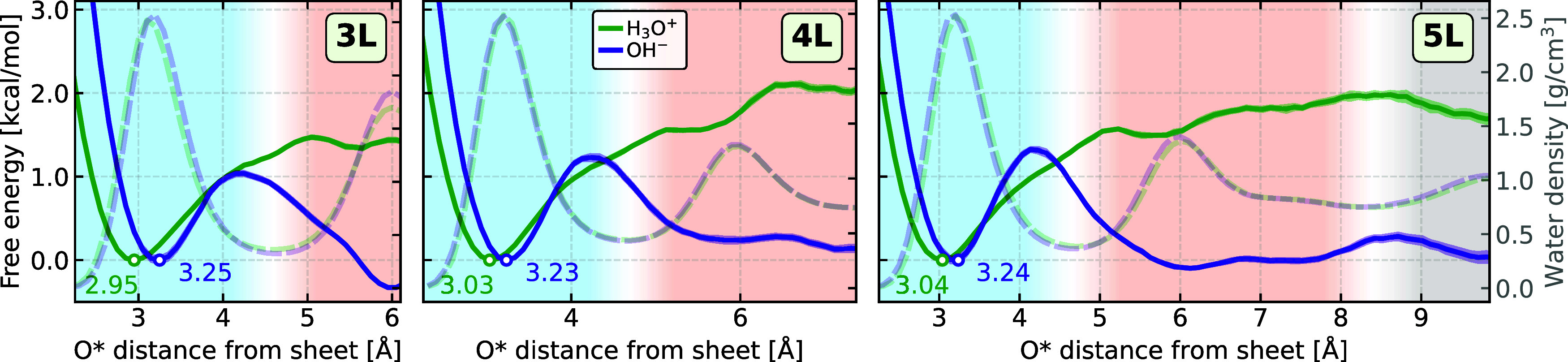
Free energy profiles and water structuring of hydronium and hydroxide near graphene sheets. Free energy profiles for the hydronium and hydroxide ions as a function of their oxygen distance O* to the closest graphene sheet obtained from the symmetrized density profiles. The minima in the interfacial layer, marked with a white dot, serve as the free energy reference point for each ion, with their distances to the interface presented. The structuring of the water layers is represented by the water density profiles, which are indicated with corresponding lighter colors and dashed lines. The shaded regions indicate the uncertainty resulting from the standard deviation of the five replicate simulations. The background’s faded blue, orange, and gray represent the interfacial, intermediate, and bulk-like water layers, respectively. The horizontal axis is displayed up to half the distance between the two layers.

Using the simulated density profiles, we quantified the surface affinity of hydronium and hydroxide ions by examining their free energy profiles. To accurately define this affinity, systems must include both interfacial and noninterfacial layers; otherwise, all layers would be considered interfacial. Therefore, we analyzed systems with an intermediate water region (i.e., 3L–5L). Our analysis focuses on the distance between the specific oxygen atom of the protonic defect (O*) and the closest graphene sheet. As shown in the free energy profiles in Figure 2, the hydronium ion is stabilized at the graphene–water interface compared to the bulk, with an energy that is substantially higher than the thermal energy, *k*_B_*T* ≈ 0.6 kcal/mol (*T* = 300 K). In contrast, the hydroxide ion exhibits a more nuanced stabilization behavior at the interface, generally showing a slight preference for layers farther from the interface. The profiles reported herein are validated using umbrella sampling to ensure effective sampling of the phase space and are explicitly compared to previous literature results^38^ (see Section S4). The results reported herein highlight the strong preference of the hydronium ion for the interface, in contrast to the hydroxide ion’s bimodal distribution, which is found both near the surface and toward the interior layers.

### Difference in Hydrogen Bonding and Orientational Behavior Explain Ion Stability

**Figure 3:**
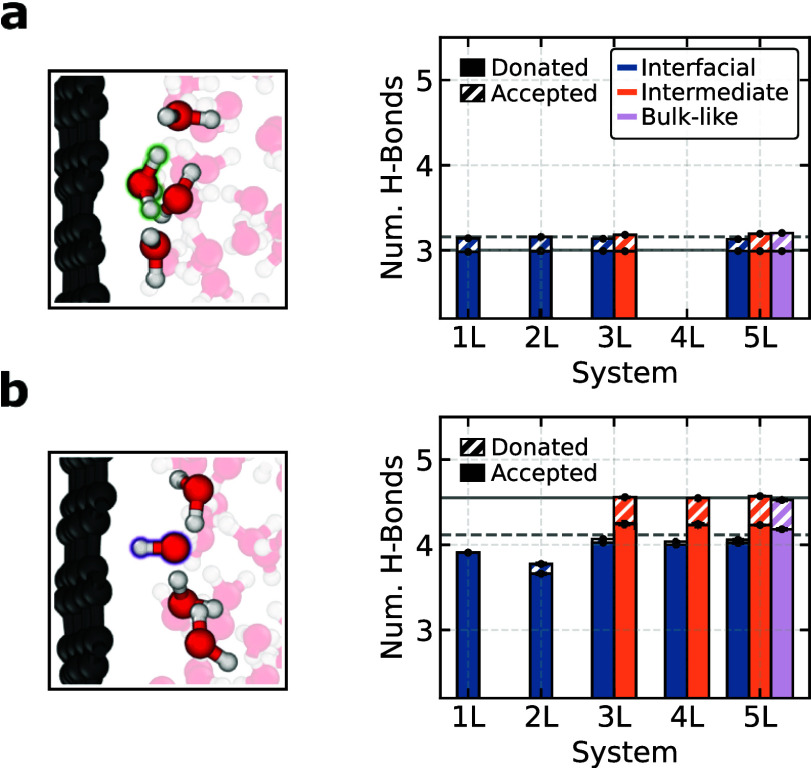
Difference in hydrogen bonding behavior of the protonic defects at the graphene–water interface. Average number of hydrogen bonds (accepted or donated, as indicated in each legend) for the hydronium ion (a) and the hydroxide ion (b) across the different water layers with their corresponding bulk reference indicated with horizontal lines. The representative snapshots show their orientational preferences at the graphene–water interface. Hydrogen bonds are counted using the geometric definition provided in ref (66). The error bars, smaller than the markers, are obtained from the standard deviation of the five replicate simulations.

To elucidate the molecular mechanism behind the observed ion behaviors, we investigate the structural characteristics of both defects, focusing on their hydrogen bonding patterns and orientational preferences at the interface. These factors are crucial for understanding their affinity for specific interfacial locations. In bulk water, the hydronium ion consistently donates three hydrogen bonds to neighboring water molecules while acting very rarely as an acceptor. Conversely, the hydroxide ion typically adopts a hypercoordinated square-planar arrangement in bulk water, accepting mostly four hydrogen bonds and transiently donating one. This arrangement, common in bulk environments, keeps the local water structure stable and enhances the ion’s stability.^19^ In contrast to these well-established bulk solvation patterns, our analysis at the interface reveals significant differences. As shown in Figure 3a, the hydronium ion maintains a stable hydrogen bond environment across all layers, consistently donating three hydrogen bonds regardless of its location. Near the interface, the hydronium ion positions its hydrogen atoms toward the water layers, lying flat as shown in the snapshot. This orientation maintains the hydrogen bond network of the water molecules in the interior layers (see Section S5) and is influenced by the hydrophobic nature of the hydronium ion’s oxygen, which typically does not accept hydrogen bonds due to its limited availability of lone-pair electrons. In contrast, as shown in Figure 3b, the hydroxide ion at the interface experiences significant hydrogen bond disruption, resulting in it accepting fewer hydrogen bonds than in bulk water and donating almost none due to spatial constraints near the interface. This disruption changes its typical orientation, with the hydroxide ion’s hydrogen atom predominantly facing the interface, impacting its usual 4-fold hypercoordinated solvation pattern and significantly reducing its stability. Overall, this analysis shows that hydronium’s hydrogen bonding is not disrupted at the interface whereas the hydroxides’ is.

### Hydronium Is Enthalpically Driven to the Interface, Hydroxide Is Entropically Driven

**Figure 4:**
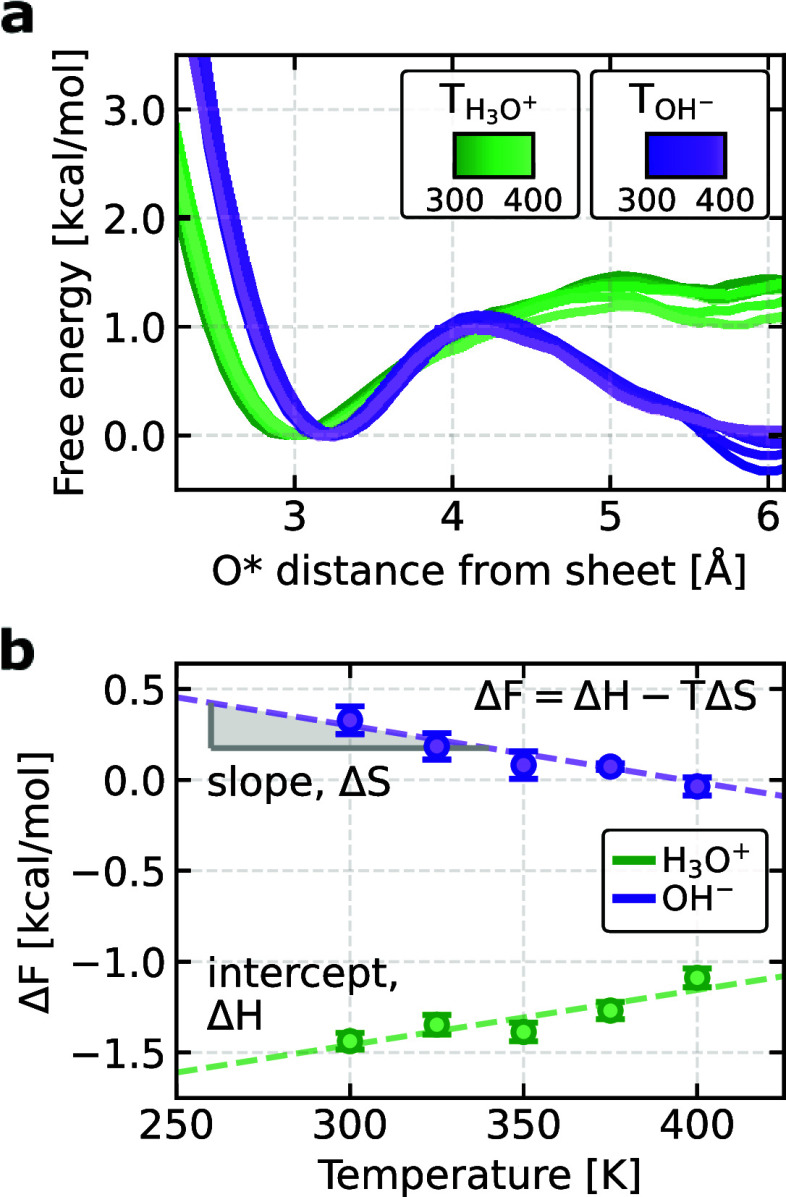
Temperature dependence of the free energy for the protonic defects. (a) Free energy profiles for the hydronium and hydroxide ions as a function of their oxygen O* distance to the closest graphene sheet at 300–400 K for the 3L system. The minima in the interfacial layers serve as the reference point for these profiles. The shaded regions represent the resulting uncertainty obtained from the standard deviation of the five replicate simulations for each temperature and protonic defect, each propagated for 2.5 ns. (b) Adsorption free energies of the hydronium and hydroxide ions to the interface as a function of temperature, along with their respective linear fits. For the hydronium ion, we obtain Δ*H* = – 2.4 ± 0.2 kcal/mol and Δ*S* = – 3.0 ± 0.6 cal/mol/K. For the hydroxide ion, we obtain Δ*H* = 1.2 ± 0.3 kcal/mol and Δ*S* = 3.1 ± 0.8 cal/mol/K. The error bars, smaller than the markers, are obtained from the standard deviation of the five replicate simulations.

We now turn our focus to the thermodynamic forces that critically influence ion stability at the interface. To investigate these forces, we observe how these ions behave within the 3L system featuring an intermediate region, across temperatures from 300 to 400 K, as shown in Figure 4a. First, we compute the adsorption free energy (Δ*F*) as the difference between the free energy at the interfacial layer and the intermediate layer of the 3L system, measured at the midpoint of the simulation box. This approach allows us to capture the thermodynamic propensity of ions to either stabilize at the interface or migrate toward more central water layers. As temperature increases, we see small but significant changes in the adsorption free energies of both protonic defects, as shown in Figure 4b. To explain these differences, we decompose the free energy into enthalpic (Δ*H*) and entropic (Δ*S*) contributions. This is done by performing a linear fit of Δ*F* = Δ*H* – *T*Δ*S*, assuming both changes are temperature-independent within this range and ignoring pressure–volume work contributions under ambient conditions. This decomposition allows us to understand the driving forces behind ion stability at the interface.

For the hydronium ion, we observe an increase in Δ*F* with temperature, thus leading to less stabilization at the interface with temperature. This highlights that entropy destabilizes the hydronium ion at the interface, while its stability comes from direct interactions with graphene and minimal disruption to the hydrogen bond network of surrounding water molecules, allowing solvent rearrangements that enhance these hydrogen bonds. This behavior is consistent with studies at the air–water interface, where hydronium is also enthalpically driven to the surface.^15,67^ However, in our system, the presence of graphene modifies the stabilization mechanism. While hydronium’s preference for the interface at the air–water boundary has been linked to a surfactant-like effect that influences the broader hydrogen-bond network, at the graphene–water interface, the stabilization of hydronium is more localized. In addition to maintaining strong hydrogen bonding with interfacial water molecules, hydronium experiences additional enthalpic stabilization through electrostatic interactions with the polarizable surface of graphene, which alters the local charge distribution. This suggests that while the thermodynamic driving force is similar to that observed at the air–water interface, the presence of graphene imposes additional changes to the interfacial environment, shifting the balance of interactions that stabilize hydronium. This primarily enthalpic interaction, inferred from the intercept of Δ*F* at the lower temperature in Figure 4b, indicates that enthalpy is the dominant contribution to the proton’s surface preference.

Conversely, the behavior of the hydroxide ion at the interface is largely influenced by entropic contributions, which increase its preference for the interface as the temperature rises. Initially, at 300 K, strong water–ion interactions retain the hydroxide ion predominantly in the bulk due to its well-defined hydration shell. However, as temperature increases, the system gains entropic stabilization from exploring the additional states of hydroxide physisorbed at the graphene interface. Unlike hydronium, hydroxide’s interfacial affinity to the graphene–water interface deviates from its behavior at the air–water interface, where it is entropically repelled.^67^ In contrast, at the graphene–water interface, hydroxide adsorption is influenced by graphene’s polarization effects, which partially stabilize hydroxide near the surface. This suggests that graphene modifies the balance of interactions governing hydroxide’s interfacial behavior when compared to purely aqueous environments. This entropic drive, reflected in the decreasing slope of Δ*F* with temperature in Figure 4b, facilitates the exchange of solvent molecules and the hydroxide ions between layers.

The observations above demonstrate that hydronium has a preference for the interface due to enthalpic forces, while hydroxide is driven by entropic forces. They also reveal the complex balance between these forces at the interface and show how temperature influences ion stability.

### Graphene Influences the Ionic Interactions at the Interface

The hydronium and hydroxide surface propensities reported herein bear similarities with those previously observed at the air–water interface, where the interface is typically described as being enriched with hydronium ions and depleted of hydroxide ions relative to the bulk.^21,68^ In our study, similar to what is observed at the air–water interface, hydronium ions are typically closer to the graphene layers than hydroxide ions, indicated by their shorter oxygen distances O* from the sheet (recall Figure 2). However, unlike the air–water interface, where capillary wave fluctuations and hydrogen bond rearrangements dominate ion stabilization,^15,69^ the graphene–water interface introduces additional polarization effects that influence ion behavior. In our system, hydronium retains a stable hydrogen bond network at the interface, similar to what is observed at the air–water interface. However, while hydronium adsorption at the air–water interface is primarily driven by hydrogen-bond network reorganization and a surfactant-like effect, at the graphene–water interface, it also experiences electrostatic stabilization from polarization effects in graphene, which modulates the local charge distribution and influences nearby hydrogen bonds. These differences are expected to have important implications for interfacial fluctuations and surface tension effects. While ions at the air–water interface modify capillary wave fluctuations, thereby impacting surface tension, graphene’s rigidity suppresses these fluctuations, reducing the contribution of capillary waves. Additionally, graphene’s polarization effects induce charge rearrangements, further stabilizing hydronium and creating a local free energy minimum for hydroxide near the surface (see Figure 2). The presence of a solid substrate also constrains large-scale water reorganization, typically observed at the air–water interface.

**Figure 5:**
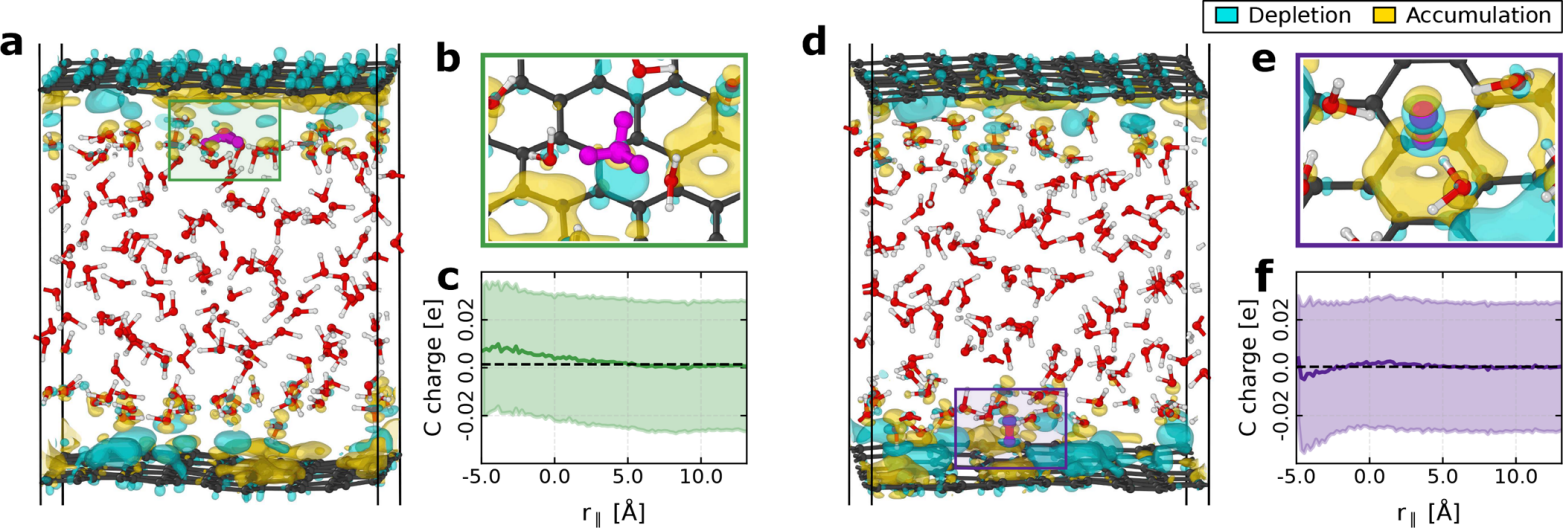
Role of graphene and its interaction with the liquid environment. Isosurfaces representing constant electron density differences for representative snapshots in the 5L acidic (a) and basic (d) systems. In panel (a), the hydronium ion is highlighted in magenta, while in panel (d), the hydroxide ion is also colored in magenta for clarity. Blue isosurfaces indicate regions of electron depletion, whereas yellow isosurfaces indicate regions of electron accumulation. The solid black lines mark the edges of the periodic simulation box. The electron density difference units are 0.75 × 10^–5^ e/Å^3^. Panels (b) and (e) provide a zoomed-in view of the local environment surrounding the hydronium and hydroxide ions, which are highlighted in panels (a) and (d), respectively. Panels (c) and (f) show the carbon charge distribution as a function of in-plane distance *r*_∥_ from a hydronium or hydroxide ion, respectively. The shaded areas represent one standard deviation, while the black dashed lines indicate the overall average carbon charge (see Section S6 for details).

To assess the significance of the interactions between the liquid environment and the graphene layers, we used DFT to analyze the electron density of the interfaces (see definition in Methods). In particular, we looked at electron density differences, aimed at capturing the rearrangement of electron density due to the interaction between graphene and the liquid environment. Key results of this analysis are shown in Figure 5a,d where it can be seen that graphene significantly alters the electron density of water molecules at the interface. This charge rearrangement—which is limited to the contact layers—therefore creates a distinct hydrogen bonding environment for protonic defects at the graphene–water interface compared to what they experience at the air–water interface. In addition, interesting local charge reorganization around the interfacial protonic defects is observed. As shown in Figure 5b, the oxygen of the hydronium ion exhibits a localized decrease in its negative charge upon interacting with graphene, while the nearby carbon atoms near O* show a slight positive polarization. This localized effect is further quantified through Bader charge analysis, with the corresponding data presented in Figure 5c (see Section S6 for further details). When the hydroxide ion points with its hydrogen toward the interface, it can induce electron accumulation above the entire C6 ring, as shown in Figure 5e. However, Bader charge analysis reveals that this is only a local effect rather than a global charge reorganization, as demonstrated in Figure 5f. This polarization effect is more subtle than that induced by the hydronium ion because the dangling hydrogen in the hydroxide ion has greater freedom in its orientation, leading to more variable polarization effects. Notably, while water molecules may occasionally orient their OH bond toward the graphene and induce similar polarization effects, this alignment is transient. In contrast, hydroxide ions consistently orient their OH group toward the graphene, resulting in a stronger and more persistent impact due to their stable interaction with the surface (see Section S6).

These findings demonstrate a clear outcome: the response of graphene to ions at the interface is counterintuitive. The cation induces charge depletion in nearby carbon atoms, while the anion induces charge accumulation. To understand this behavior it is crucial to consider their asymmetrical charge arrangements and preferred orientations at the interface. This intriguing finding emphasizes the need to carefully consider these intricate interactions when interpreting surface experiments at the graphene–water interface, such as zeta potential measurements.

## Conclusions

Our findings demonstrate a clear preference for the hydronium ion at the graphene–water interface under various confinement regimes, rendering the graphene–water interface enriched with hydronium ions. Enthalpic forces drive the hydronium ion to the interface, maintaining the hydrogen bonding network and enabling energetically favorable interactions with graphene. Conversely, a subtle balance of entropic and enthalpic contributions generally pushes the hydroxide ion toward the bulk at ambient conditions, despite a clear local free energy minimum in the first contact layer, supporting its preference for optimal water interactions. In summary, our work emphasizes the complex interplay of hydrogen bonding, orientational preferences, and thermodynamic forces that dictate the stability of hydronium and hydroxide ions at the graphene–water interface. Coupled with surface polarization effects, leading to counterintuitive charge rearrangements within graphene, these findings provide a comprehensive picture of the two species under confinement and at the graphene–water interface. Importantly, these findings were made possible by leveraging machine learning-based MD simulations of systems encompassing up to 700 atoms and spanning over 200 ns while retaining first-principles accuracy, far beyond the limits set by AIMD. This approach is crucial to our study as it allows us to achieve DFT-level accuracy and extensively sample the phase space, providing insights into the microscopic mechanisms driving these interactions.

The mechanistic insights provided in this work are expected to significantly influence and guide future experimental studies. Previous work^39^ has rationalized the macroscopic experimental behavior of nanoconfined electrolytes by postulating the presence of a negative surface charge due to the adsorption of hydroxide ions at the carbon interface. However, our findings not only demonstrate a lack of preferential hydroxide adsorption but also a complex relationship between ion adsorption and surface charge, wherein an adsorbed hydroxide can negatively polarize the surface. In real-world conditions, additional factors such as the presence of bicarbonate ions,^70^ hydrophobic impurities,^71^ or hydrocarbon adsorption^72^ may further influence the zeta potential of graphene and other nonionizable hydrophobic surfaces. These species influence interfacial charge distributions, modify local electrostatics, and affect the hydration structure at the interface, complicating a direct attribution of surface charge to hydroxide adsorption alone. Importantly, the mechanisms governing these adsorption behaviors—hydrogen bonding and ion orientational effects—cannot be captured by continuum theories like Poisson–Boltzmann which are conventionally applied to model these systems. Additionally, the ambiguity in defining interfacial depth can lead to conflicting outcomes, complicating accurate assessments of whether hydronium or hydroxide ions prefer the interface.^14^ This underscores the necessity of integrating our simulation findings with experiments that offer an atomistic resolution of interfacial signatures.^73^ Surface-sensitive spectroscopy techniques, such as second harmonic generation and sum frequency generation spectroscopies,^21,23,74,75^ provide molecular-level insights into interfacial water structure and ion distributions, as they are active only in noncentrosymmetric environments. Single-molecule localization microscopy has also been used to track proton transport at the hexagonal boron nitride-water interface, revealing a preferential lateral transport pathway.^76^ Applying similar methodologies to graphene interfaces could further clarify interfacial proton dynamics and charge transport in carbon-based systems. In addition, nuclear magnetic resonance spectroscopy and pH measurements within porous carbon can provide valuable insights into the local ion environment and interfacial charge distributions.^77^ These techniques, when combined with atomistic-resolution approaches, help bridge the gap between microscopic simulations and experimental observables. Finally, direct atomic resolution imaging via atomic force microscopy could further elucidate differences in water structure,^78^ offering a deeper understanding of how these interactions influence conductivity in graphene.

This study provides a detailed picture of protonic defects at the graphene–water interface as a foundation for interpreting experimental data and advancing our fundamental understanding of ions at interfaces. Our prediction of high proton propensity to the graphene–water interface opens up the possibility for technological innovations in nanofluidics, heterogeneous solid–liquid catalysis, and other critical domains that rely on proton-mediated processes. Finally, given the model system character of our setup, we anticipate that this proton-enriched interface behavior may be transferable to other systems like water in biological channels, geological formations, and technological nanodevices.

## Methods

### Machine Learning Potentials

In this work, we use the MACE architecture,^53^ which allows for fast and highly data-efficient training with high-order equivariant message passing and has been proven robust in a wide variety of scenarios.^79^ We developed and validated a MACE MLP model (see Section S2) with two layers, a 6 Å cutoff distance, 128 equivariant messages, and a maximal message equivariance of *L* = 1. The MLP captures semilocal interactions through a receptive field that spans the product of the number of layers and the cutoff distance per layer. In this case, the total receptive field is 12 Å, allowing the MLP to account for interactions within this range. While the model does not explicitly account for long-range effects, the 12 Å receptive field spans nearly the entire width of the slit in most cases, effectively capturing the relevant electrostatic interactions within the simulation. The final energy and force validation root-mean-square errors were 0.7 meV/atom and 17.2 meV/Å, respectively.

To accurately represent the potential energy surface of the systems, we train our MLP model using energies and atomic forces obtained from DFT calculations using the CP2K/Quickstep code.^80^ We specifically used the revPBE-D3^54,55^ functional due to its robust performance in reproducing the structure and dynamics of liquid water,^56−58^ while also effectively capturing protonic defects^60^ and the interaction energies between water and graphene.^59^ This accuracy partly stems from a fortuitous cancellation between errors in the functional and the neglect of nuclear quantum effects (NQEs). A more detailed investigation of NQEs and their influence on the confined nanoscale dynamics of excess protons and hydroxide ions remains an important avenue for future work. However, we emphasize that the surface affinity shows no significant dependence on temperature or functional choice, giving us confidence that NQEs will not affect the main conclusions of this study. Atomic cores are represented using dual-space GTH pseudopotentials.^81^ The Kohn–Sham orbitals of oxygen and hydrogen atoms are expanded using the TZV2P basis set, while those of carbon atoms are expanded using the DZVP basis set. An auxiliary plane-wave basis with a cutoff of 1200 Ry was used to represent the density. We have examined the influence of the functional and dispersion correction on our results and found that the observed trends remain consistent, irrespective of these choices (see Section S2). For interfacial systems, we used a vacuum of 15 Å to uncouple periodic images in the *z* direction, leading to negligible interactions between the images as confirmed by the convergence of the energy with respect to the vacuum size. See Section S1 for further details.^82^

The MLP model was systematically developed over five generations. The first generation involved a training set obtained from previous work,^36^ enhanced by an active learning procedure^47,83^ to incorporate structures that explicitly account for water–carbon interactions at slit widths of 5 and 6.5 Å. This included conditions ranging from low- to high-density water at various temperatures, including 100, 300, and 600 K. The second generation incorporated structures obtained from path integral MD simulations to capture the quantum fluctuations of the nuclei in our model. For the third generation, we targeted various slit widths, including 6, 10, 15, and 20 Å, and included structures corresponding to these dimensions. In the fourth generation, we conducted an additional round of active learning to refine the model based on the conditions sampled thus far. This led to the fifth generation, which included configurations of neutral frames containing a protonic defect pair (both a hydronium and a hydroxide ion) under both bulk water conditions and confined conditions at slit widths of 6, 10, 13 Å. To avoid the complications of applying a background charge for charge neutrality, we did not include isolated protonic defects as suggested in ref (60). The final model consisted of 3378 structures, with 1303 involving graphene interfaces and 2075 associated with bulk conditions.

### Molecular Dynamics Simulations

All MD simulations reported herein, which were based on the MLP, were performed using the ASE software^84^ at a temperature of 300 K, unless explicitly stated otherwise, in the *NVT* ensemble. A time step of 0.5 fs was employed, and simulations utilized a Langevin thermostat with a friction coefficient of 2.5 ps^–1^. For each of the five slit widths and two species, we conducted five independent simulations. Each simulation included a 90 ps equilibration period followed by a 4 ns production run, resulting in a total of over 200 ns of simulation time. Uncertainties in reported values were calculated using the standard deviation from these replicates. All systems were simulated in orthorhombic simulation cells employing periodic boundary conditions in all three directions. The simulation cells were initially set up by randomly packing several molecules between the graphene sheets to form one to five well-defined layers of water. To prevent interactions between the periodic images, 15 Å vacuum (exceeding the model’s receptive field) was added in the *z* direction of these initial configurations. To achieve equilibrium density, the graphene sheets were treated in a fully flexible manner, allowing them to adapt without additional constraints. To validate the findings reported, we conducted additional MLP-based biased simulations using umbrella sampling to compare the free energy profiles reported. See Sections S1 and S4 for further details.

### Electronic Structure Analyses

The electronic properties of the protonic defects at the graphene–water interface were analyzed using the same electronic structure settings used to train our MLP. However, to reduce the computational cost, a cutoff of 1050 Ry was used. To assess the interactions between the liquid environments and the graphene layers, we used DFT to analyze their electron density difference (Δρ), defined as Δρ = ρ_liq/gra_ – ρ_gra_ – ρ_liq_, where ρ_liq/gra_, ρ_gra_, and ρ_liq_ are the electron densities of the system under consideration, the isolated graphene surfaces, and the isolated liquid environment, respectively. Where appropriate, the system’s net charge was neutralized using a uniformly charged background. To determine the response of graphene to the protonic defects at the interface, we performed Bader charge analysis using the same settings reported herein with the reduced cutoff (see Section S6).

## Data Availability

All data required to reproduce the findings of this study is available at (https://github.com/water-ice-group/graphene-water-protons).

## Supporting Information Available

Additional details on system setup; validation of the machine learning potential; assessment of the effect of graphene flexibility; details on the potential of mean force calculation with umbrella sampling; additional details on the water hydrogen bonding within the systems; and supporting partial charge analysis (PDF)
