# Supplementary material for: Protons Accumulate at the Graphene–Water Interface
Source: ACS Nano. 2025 Apr 28;19(18):17728–37. doi: 10.1021/acsnano.5c02053 (PMC12080325; doi:10.1021/acsnano.5c02053)
Supplement: Supplementary file 1 — nn5c02053_si_001.pdf [file nn5c02053_si_001.pdf]

Supporting Information: Protons accumulate at the graphene-water interface

Xavier R. Advincula,[†,‡,¶] Kara D. Fong,[*,†,¶] Angelos Michaelides,[*,†,¶] and Christoph Schran[*,‡,¶]

†*Yusuf Hamied Department of Chemistry, University of Cambridge, Lensfield Road, Cambridge, CB2 1EW, UK*

‡*Cavendish Laboratory, Department of Physics, University of Cambridge, Cambridge, CB3 0HE, UK*

¶*Lennard-Jones Centre, University of Cambridge, Trinity Ln, Cambridge, CB2 1TN, UK*

E-mail: kdf22@cam.ac.uk; am452@cam.ac.uk; cs2121@cam.ac.uk

Contents

1 Molecular dynamics simulations **S-3**

1.1 System setup . . . S-3

1.2 Identification of hydronium and hydroxide ions . . . S-7

1.3 Simulation setup . . . S-7

2 Machine learning potential **S-10**

2.1 Model development . . . S-10

2.2 Model validation . . . S-10

3 Effect of graphene flexibility **S-25**

4 Potential of mean force calculation with umbrella sampling **S-27**

5 Water hydrogen bonding **S-31**

6 Partial charge analysis **S-32**

References **S-34**

1 Molecular dynamics simulations

1.1 System setup

The systems studied are labeled as 1L, 2L, 3L, 4L, and 5L corresponding to systems with two parallel free-standing graphene sheets (each containing 112 atoms) separated by heights ranging from approximately 6.5 to 20 Å and intercalated by one (1L), two (2L), three (3L), four (4L), and five of water (5L). These different slit widths correspond to varying amounts of water molecules corresponding to system sizes between ≈ 300 to 700 atoms. The initial slit widths considered were 6.91 Å, 9.41 Å, 12.20 Å, 14.41 Å, and 19.41 Å. In each system, the graphene sheets have dimensions $L_x = 17.290$ Å and $L_y = 17.112$ Å, derived by repeating the base unit cell dimensions of $a = \sqrt{3}d_c$ and $b = 3d_c$ multiple times along the x and y directions, respectively. Specifically, L_x corresponds to approximately seven repetitions of the unit cell dimension a along the x-axis, while L_y is the result of four repetitions of the unit cell dimension b along the y-axis. This tiling of the unit cell ensures that the graphene sheet maintains its characteristic hexagonal lattice structure with a consistent carbon-carbon bond distance of $d_c = 1.42$ Å [S1] throughout the extended sheet. All systems were simulated in orthorhombic simulation cells employing periodic boundary conditions in all three directions. To prevent interactions between the periodic images, a vacuum space of 15 Å vacuum was added in the z direction of the initial configurations. In the MACE architecture, each layer interacts only with neighboring atoms within a specified cutoff distance, which sets the interaction range for that layer. As information is passed through successive layers, the receptive field expands to include neighbors at increasing distances, theoretically extending up to the product of the number of layers and the cutoff. However, when a vacuum is present and no atoms fall within this cutoff, message-passing is restricted, effectively limiting the receptive field to the local cutoff of 6 Å. Thus, the 15 Å vacuum lies beyond the model's effective receptive field and the energy convergence threshold of our electronic structure settings, ensuring decoupling in the z-direction. An overview of the systems studied,

including a hydronium or hydroxide ion, is provided in Fig S1 and Table S1.

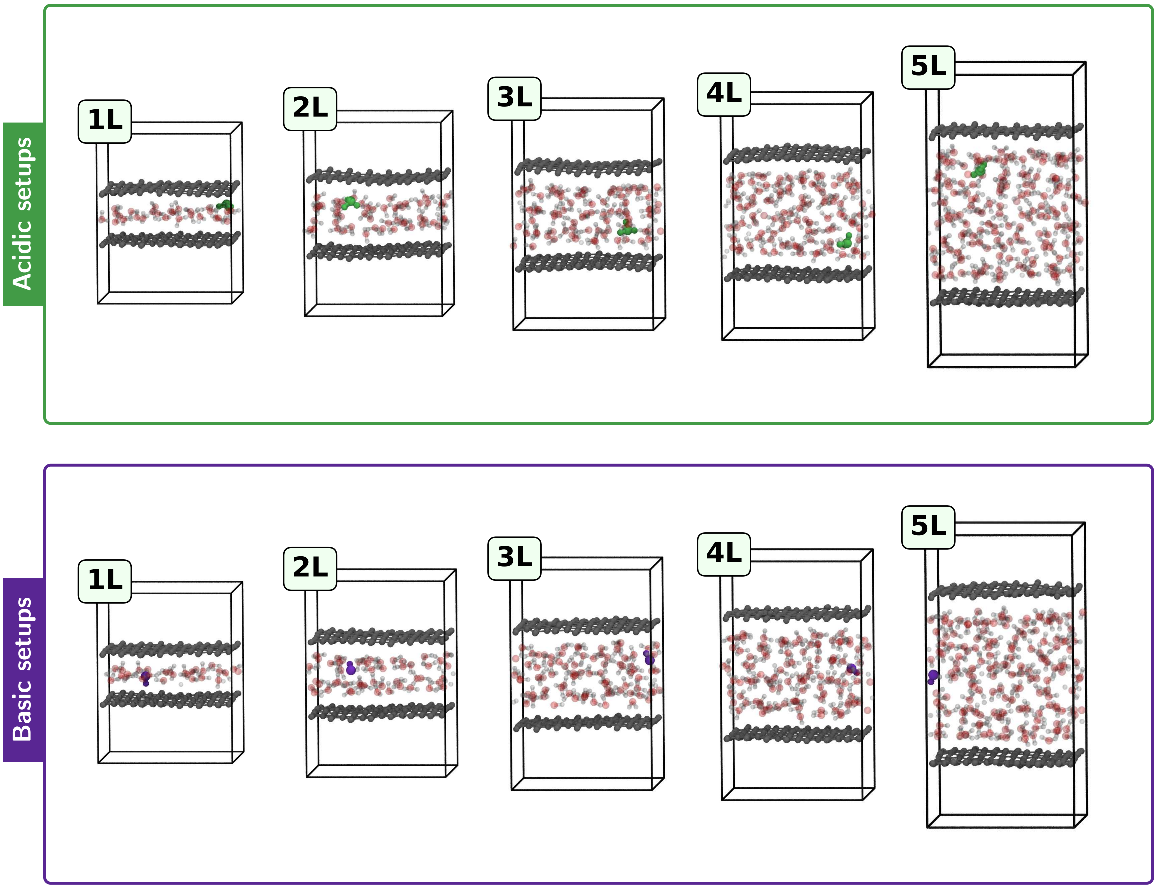

Figure S1: Representative configuration snapshots of the systems studied in this work. In the acidic setups, the hydronium ion is represented in green, while in the basic setups, the hydroxide ion is represented in purple. The solid black lines indicate the edges of the periodic simulation box.

The graphene sheets in our simulations were treated in a fully flexible manner to achieve the corresponding equilibrium density. The simulation cells were initially prepared by randomly packing molecules between the graphene sheets to form one to five distinct layers of water. The initial quantity of water molecules was chosen based on prior studies[S2,S3] to achieve slit widths comparable to experimentally realizable pore dimensions. To provide a sense of the system size, Fig. S2 shows the average slit width and its fluctuations over a 500 ps period for each run.

Our simulation setups are particularly well-suited for experimental comparison, as they avoid artificial pressures that could alter the natural behavior of confined water. By allowing the graphene sheets to remain fully flexible and reach equilibrium density under "zero pressure" conditions, we capture the intrinsic properties of water confined between graphene layers without imposing external constraints that might complicate experimental replication. This design makes our results directly comparable to experimental setups, where slit pores are often constructed with graphene-graphene distances close to those in our model. Notably, our selected pore distances align well with experimentally achievable channels, such as those reported in Ref. S4, and support validation through techniques like sum-frequency generation vibrational spectroscopy, which has been applied to study graphene-water interfaces in Ref. S5.

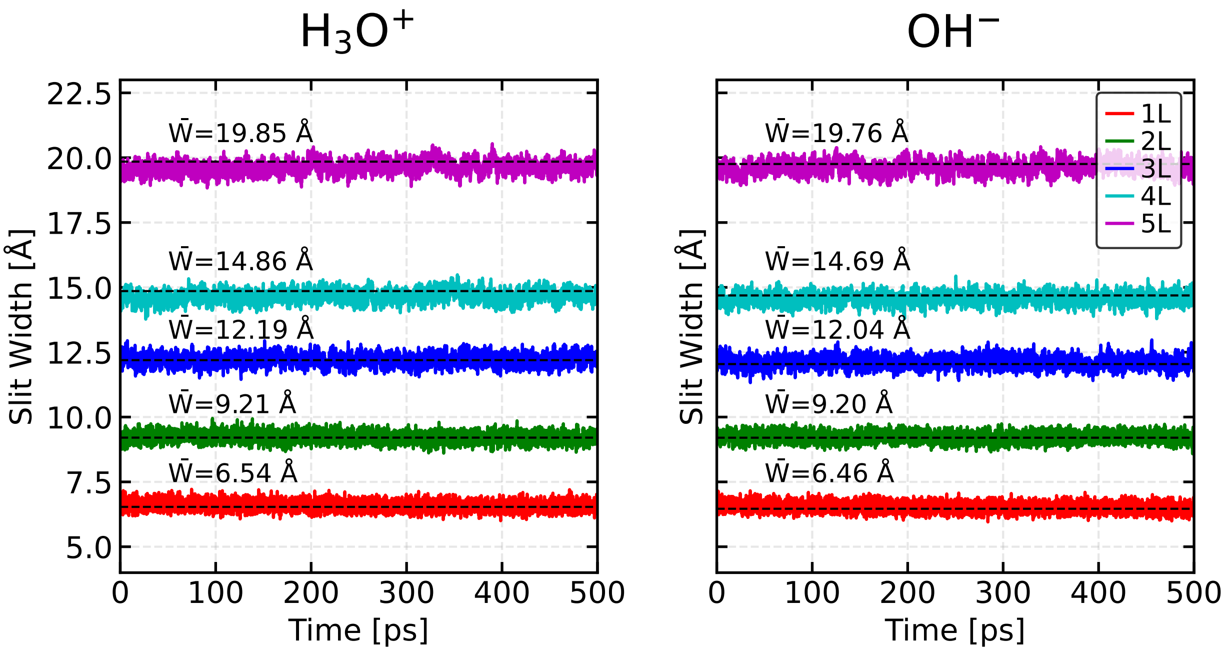

Figure S2: Fluctuations in the slit width of the systems studied here for both the acidic (left) and basic (right) setups. For each slit width and each species, the average slit widths $\bar{H}$ over an entire run are reported to illustrate the size of the systems studied.

Table S1: Overview of both the acidic and basic setups. In each system, the graphene sheets have dimensions $L_x = 17.290$ Å and $L_y = 17.112$ Å . For each system, we report their average slit width, $\bar{W}$; the total number of atoms, N_{atoms}; the corresponding number of water molecules $N_{\text{H}_2\text{O}}$ and protonic defects (i.e., either a hydronium ion $N_{\text{H}_3\text{O}^+}$ or hydroxide ion N_{OH^-}); the number of replicate runs, N_{runs}; the equilibration time, t_{eq}; and the simulation production time, t_{sim}.

System		1L	2L	3L	4L	5L
Acidic setups	$\bar{W}$ [Å]	6.54	9.21	12.19	14.86	19.85
	N_{atoms}	309	378	471	549	699
	$N_{\text{H}_2\text{O}}$	27	50	81	107	157
	$N_{\text{H}_3\text{O}^+}$	1	1	1	1	1
	N_{runs}	5	5	5	5	5
	t_{eq} [ps]	90	90	90	90	90
	t_{sim} [ns]	4	4	4	4	4
Basic setups	$\bar{W}$ [Å]	6.46	9.20	12.04	14.69	19.76
	N_{atoms}	307	376	469	547	697
	$N_{\text{H}_2\text{O}}$	27	50	81	107	157
	N_{OH^-}	1	1	1	1	1
	N_{runs}	5	5	5	5	5
	t_{eq} [ps]	90	90	90	90	90
	t_{sim} [ns]	4	4	4	4	4

1.2 Identification of hydronium and hydroxide ions

The hydronium and hydroxide ions do not have a static structure; therefore, their identification requires dynamic identification in each configuration. Here, we follow the definitions proposed in Ref. S6. To recognize the hydronium ion, we initially assigned the two closest hydrogen atoms to each oxygen atom. Subsequently, the remaining unassigned hydrogen was linked to its nearest oxygen, which we identified as the oxygen of the hydronium ion, denoted as O^*.

Similarly, the identity of the hydroxide ion also changes throughout the simulation due to proton transfer events. To determine the oxygen of this anionic defect, we again assigned the two nearest hydrogen atoms to each oxygen. Next, we identified the hydrogen that was assigned to two oxygens and reassigned it to the oxygen to which it was closest. The oxygen left with only one hydrogen is then recognized as the hydroxide ion's oxygen, O^*.

1.3 Simulation setup

In this work, we conducted four different types of molecular dynamics (MD) simulations: (i) short *ab initio* MD (AIMD) simulations to generate training data for the development of the MACE machine learning potential (MLP); (ii) additional AIMD simulations to generate reference data for the validation of the MLP; (iii) extensive unbiased MD simulations using the developed MLP, comprising the main results reported in this work; and (iv) additional MLP-based biased simulations using umbrella sampling to compare the free energy profiles reported. All simulations involved a flexible treatment of the graphene sheets and employed hydrogen atoms unless explicitly stated otherwise.

Short AIMD simulations

The AIMD simulations were performed using the CP2K/Quickstep code[S7] in the NVT ensemble with a time step of 1 fs. The temperatures were set at 100, 300, and 600 K and maintained using a combination of a CSVR thermostat[S8] and an adaptive Langevin thermostat.[S9] The revPBE generalized gradient approximation exchange-correlation functional[S10]

with the zero-damping variant of the Grimme's D3 dispersion correction[S11] was used, in combination with the dual-space Goedecker-Tetter-Hutter pseudopotentials[S12] to represent the atomic cores, a 450 Ry plane wave cutoff, and the TZV2P basis set to expand the Kohn-Sham orbitals of oxygen and hydrogen atoms or the DZVP basis set to expand those of carbon atoms.[S13] To maintain stable simulations with a computationally manageable time step, deuterium masses were used. For the final training of the MLP, all the training structures were reevaluated by performing single-point density functional theory (DFT) calculations with an increased plane wave cutoff of 1200 Ry.

Reference AIMD simulations

The reference AIMD simulations to validate the MLP were performed using the CP2K code[S7] in the NVT ensemble with a time step of 1 fs. The temperature was set to 300 K and maintained using a CSVR thermostat[S8] with a 30 fs coupling constant. To maintain stable simulations with a computationally manageable time step, deuterium masses were used. A 15 ps equilibration period was followed by a 150 ps production period.

Unbiased MLP-based MD simulations

The unbiased MLP-based MD simulations were performed using ASE[S14] in the NVT ensemble where the temperature was maintained at 300 K, unless explicitly stated otherwise. For this, a Langevin thermostat with a friction coefficient of 2.5 ps^{-1} was used. The timestep was set to 0.5 fs. For each slit width and protonic defect, we conducted five replicate runs involving a 90 ps equilibration period followed by a 4 ns production period, from which statistics of the observables of interest were sampled. The equilibration period consisted of an initial 45 ps phase during which the graphene sheets were fully immobilized to equilibrate the water molecules with the protonic defects. This was followed by a subsequent 45 ps phase where the graphene sheets were treated as fully flexible, ensuring thorough equilibration.

Biased MLP-based MD simulations

The additional biased MLP-based MD simulations were performed using the LAMMPS simulations package[S15] with the PLUMED plugin[S16] in the NVT ensemble where the tem-

perature was maintained at 300 K. For this, a Nosé-Hoover thermostat with a damping constant of 0.05 ps was used. The timestep was set to 0.5 fs. For each system, a 40 ps equilibration period was followed by a 75 ps production period, from which statistics of the observables of interest were sampled. The equilibration period comprised an initial phase of 20 ps with the graphene sheets fully immobilized to properly equilibrate the water molecules with the protonic defects, followed by a subsequent 20 ps phase where the graphene sheets were treated as fully flexible.

2 Machine learning potential

2.1 Model development

The MLP model was progressively developed over five generations. The initial generation leveraged a training set from prior work[S17] and incorporated an active learning approach[S18,S19] to integrate structures designed for water-carbon interactions at slit widths of 5 and 6.5 Å. This stage accommodated a spectrum of conditions from low- to high-density water at temperatures including 100, 300, and 600 K. The second iteration introduced structures from path integral MD simulations, capturing the quantum nature of nuclei. In the third generation, the model was expanded to include various slit widths –6, 10, 15, and 20 Å– adding structures tailored to these dimensions. The fourth iteration involved another round of active learning to refine further the model based on previously analyzed conditions. Finally, the fifth generation integrated configurations containing both a hydronium and a hydroxide ion at slit widths of 6, 10, and 13 Å in bulk water. This choice deliberately avoided the inclusion of isolated protonic defects to circumvent the need for external charges for maintaining charge neutrality, as suggested in Ref. S20. This approach prevents variations in box energies that could arise from different box volumes, thus enhancing the stability and reliability of the simulation results.

2.2 Model validation

Energy and force errors

To quantify the root-mean-square error (RMSE) of the energies and forces predicted by the MLP, we conducted a detailed analysis using structures generated from 500 ps MLP-based MD simulations at 300 K. These simulations covered various slit widths and both types of protonic defects. From these simulations, 100 snapshots were randomly selected for each setup, and their energies and forces were calculated using single-point DFT calculations. In cases where the system contained a hydronium or hydroxide ion, a homogeneous background

charge was applied to maintain charge neutrality. The energies predicted by the MLP were then adjusted by subtracting a constant energy offset to account for the shift introduced by the homogeneous background charge. To mitigate the high computational costs typically associated with electronic structure calculations on large systems, we scaled down the size of the systems by setting the dimensions of the graphene sheets to $L_x = 12.350$ Å and $L_y = 12.834$ Å. This validation approach is particularly robust as it assesses structures derived directly from the MLP's potential energy surface, employed in the active learning protocol to develop the model. As seen in Figs. S3 and S4, there is an excellent agreement between the MLP and the results with the underlying level of theory, demonstrating the MLP model's ability to effectively reproduce the energies and forces obtained from the reference DFT calculations.

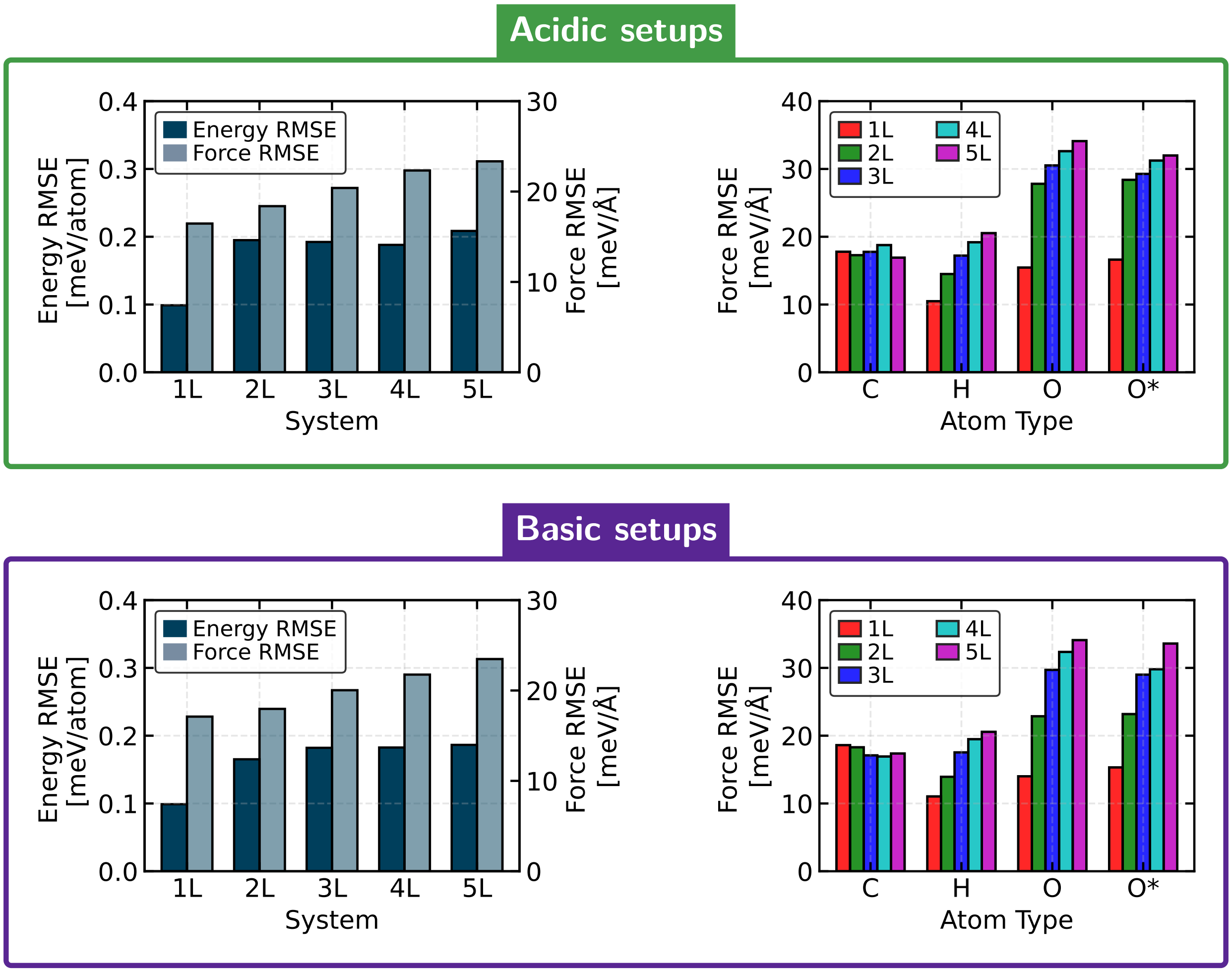

Figure S3: RMSE of the energies and forces obtained using the MLP at 300K, compared to the reference DFT calculations across the five slit widths (left), along with their force RMSE broken down by different atom types, including the specific oxygen of the protonic defect O* (right), for both the acidic and basic setups.

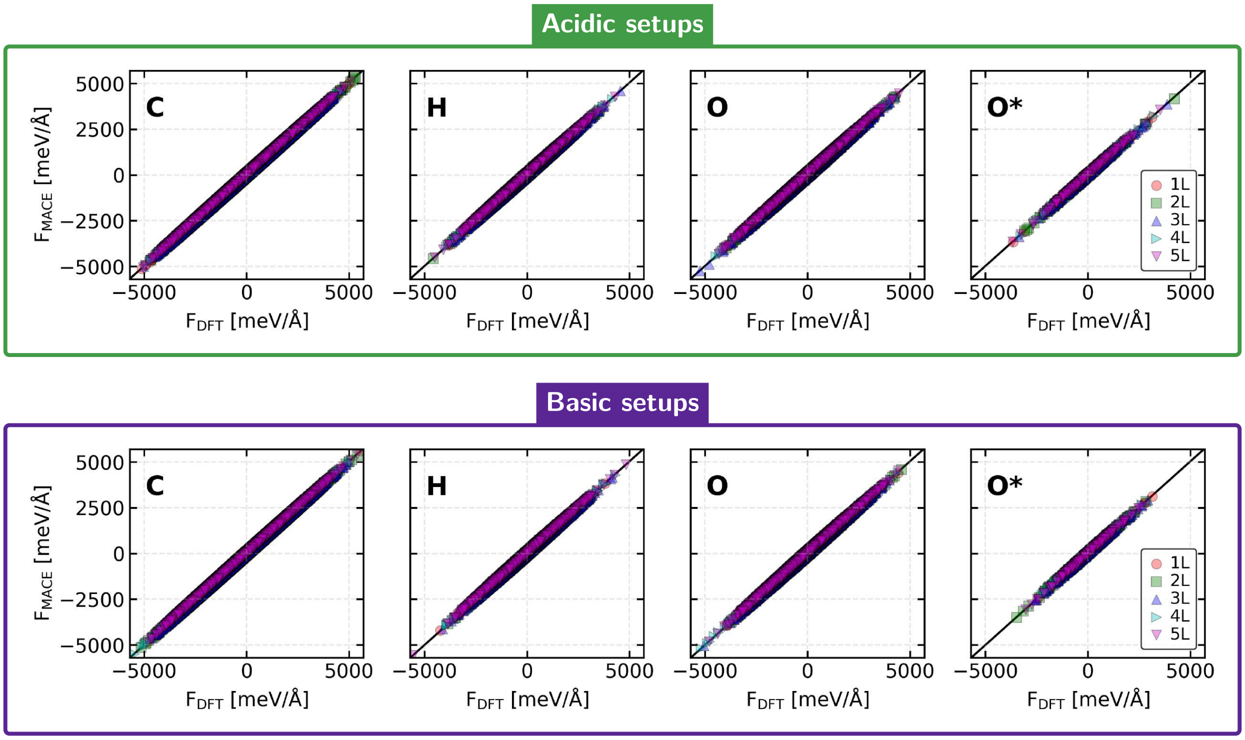

Figure S4: Parity plots for the forces obtained using the MLP, compared to the reference DFT calculations, broken down by different atom types including the specific oxygen of the protonic defect O*, across the five slit widths for both the acidic and basic setups.

Since the model is also used to simulate conditions across a temperature range from 300 to 400 K, we conducted similar analyses to those presented above for additional temperatures of 325, 350, 375, and 400 K. This is presented in Fig. S5.

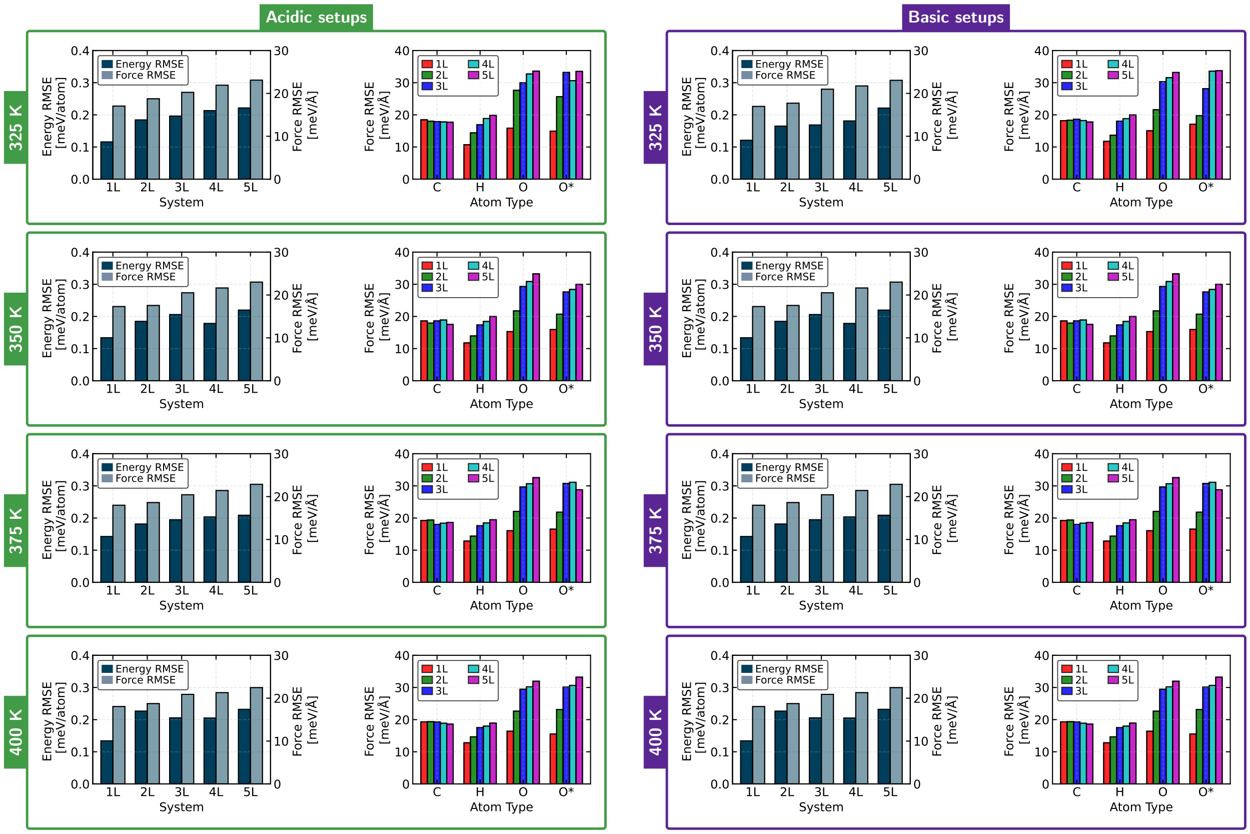

Figure S5: RMSE of the energies and forces obtained using the MLP at 325, 350, 375, and 400 K, compared to the reference DFT calculations across the five slit widths (left), along with their force RMSE broken down by different atom types, including the specific oxygen of the protonic defect O* (right), for both the acidic and basic setups.

To further validate the MLP, we evaluated its performance as a function of distance from the interface, given our focus on surface affinities and interfacial phenomena. As shown in Figs. S6 and S7, the MLP accurately reproduces the underlying DFT reference across varying distances from the interface, demonstrating its robustness. The deviations observed near the graphene sheets in some systems stem from statistical noise due to the number of structures sampled, rather than a deficiency of the model.

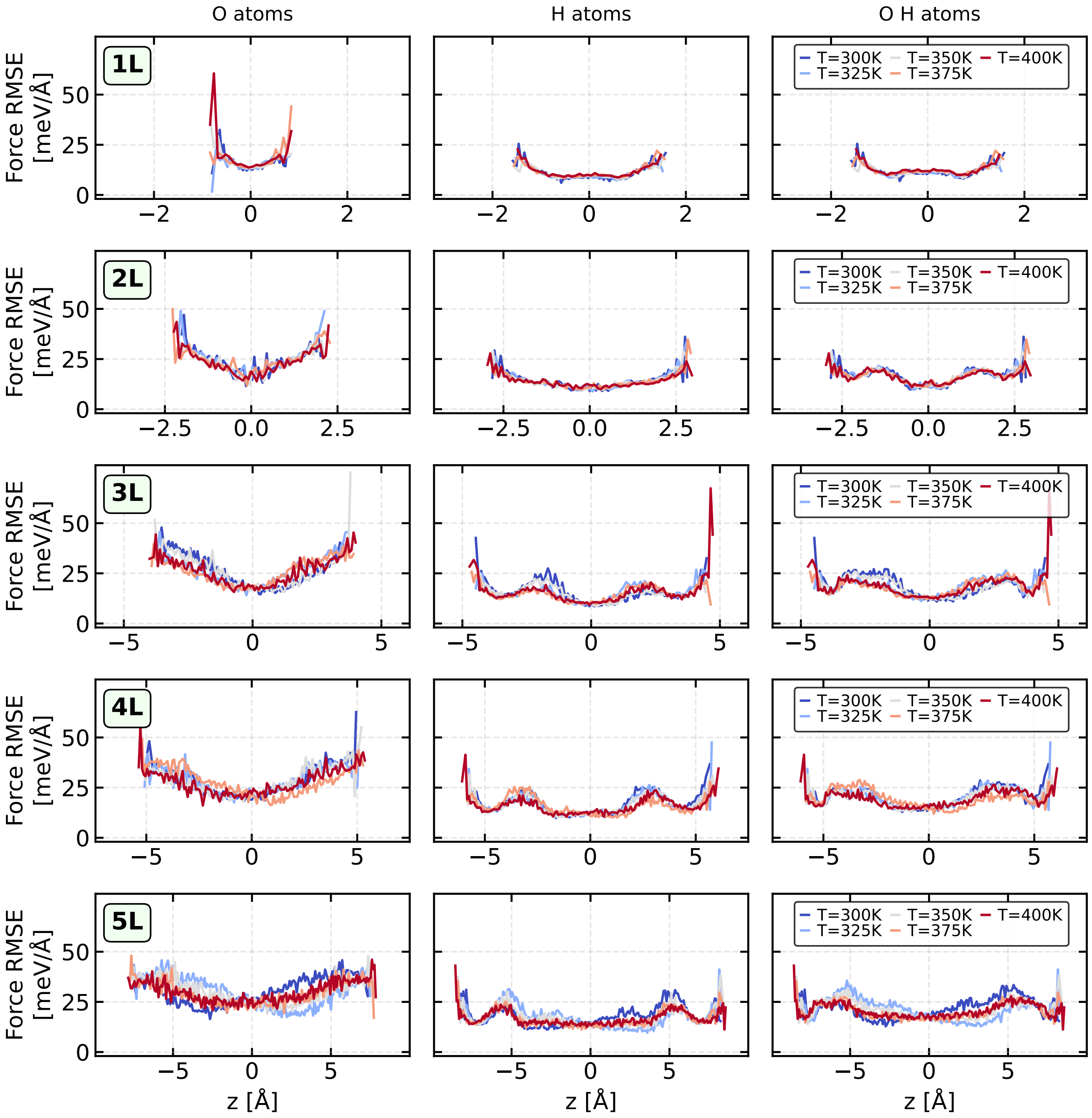

Figure S6: RMSE of the force predictions obtained using the MLP for hydronium (H_3O^+) at the graphene-water interface across different confinement regimes. Each row corresponds to a different slit width (1L to 5L), while each column represents the RMSE for oxygen atoms, hydrogen atoms, and both oxygen and hydrogen atoms. The colored lines denote results at different temperatures. The horizontal axis limits in each plot correspond to the average carbon layer positions.

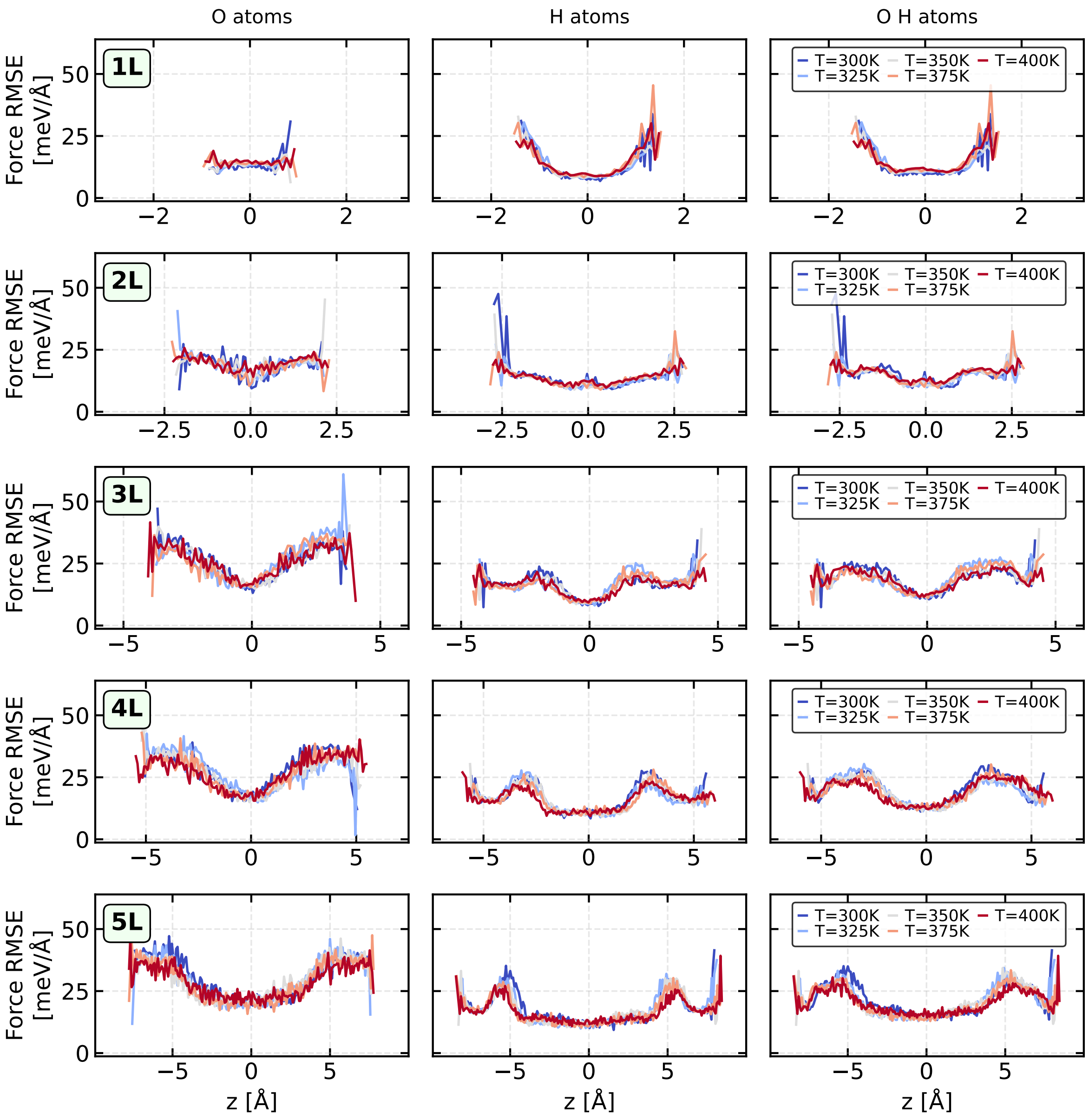

Figure S7: RMSE of the force predictions obtained using the MLP for hydronium (OH^-) at the graphene-water interface across different confinement regimes. Each row corresponds to a different slit width (1L to 5L), while each column represents the RMSE for oxygen atoms, hydrogen atoms, and both oxygen and hydrogen atoms. The colored lines denote results at different temperatures. The horizontal axis limits in each plot correspond to the average carbon layer positions.

For completeness, we also report that our MLP effectively captures temperature-induced density changes, resulting in an expansion of the water region in our setups, as shown in Table S2.

Table S2: Overview of the water region expansion of the 3L system as a function of temperature. For both the acidic and basic conditions, we report the temperature, T; the average slit width, $\bar{\mathrm{W}}$; and the standard deviation of the slit width, $\sigma_{\bar{\mathrm{W}}}$, across the different replicate runs, N_{runs}.

System	T [K]	$\bar{\mathrm{W}}$	$\sigma_{\bar{\mathrm{W}}}$	N_{runs}	t_{eq} [ps]	t_{sim} [ns]
Acidic 3L setup	300	12.194	0.200	5	90	2.5
	325	12.314	0.208	5	90	2.5
	350	12.453	0.222	5	90	2.5
	375	12.563	0.231	5	90	2.5
	400	12.723	0.261	5	90	2.5
Basic 3L setup	300	12.043	0.197	5	90	2.5
	325	12.163	0.200	5	90	2.5
	350	12.295	0.209	5	90	2.5
	375	12.413	0.220	5	90	2.5
	400	12.538	0.236	5	90	2.5

Comparison to reference AIMD simulations

To further validate the MLP, we generated AIMD trajectories for water with protonic defects under both bulk and confined conditions. This step was crucial for benchmarking structural and dynamical properties, particularly focusing on radial distribution functions (RDFs) and the free energy profiles associated with the transfer of protonic defects. Ensuring an accurate representation of proton transfer (PT) is key to the reliability of our model.

In the bulk simulations, we used 63 water molecules and 1 protonic defect (hydronium or a hydroxide ion). For the confined simulations, graphene sheets with dimensions of 12.35 Å and 12.834 Å were used to create a 3L system. For each of these conditions, 200 ps long trajectories were produced to gather validation data. As seen in Figs. S8 and S9, there is excellent agreement between the MLP and AIMD results, underscoring the MLP model's ability to faithfully reproduce the structural and dynamical predictions of the reference DFT calculations and effectively capture the critical physics of protonic defect behavior in both bulk and nanoconfined environments. The small differences observed in the PT profiles between the MLP and AIMD are likely due to the incomplete convergence of the AIMD profiles, given the relatively short trajectory length ($\sim$120 ps). Similar discrepancies between MLP and AIMD predictions have also been reported in Ref. S20 for bulk conditions, highlighting the challenge of benchmarking MLPs against computationally expensive AIMD simulations for such dynamic properties. This further highlights the advantage of MLPs in enabling long trajectory sampling, which is essential for achieving well-converged free energy profiles.

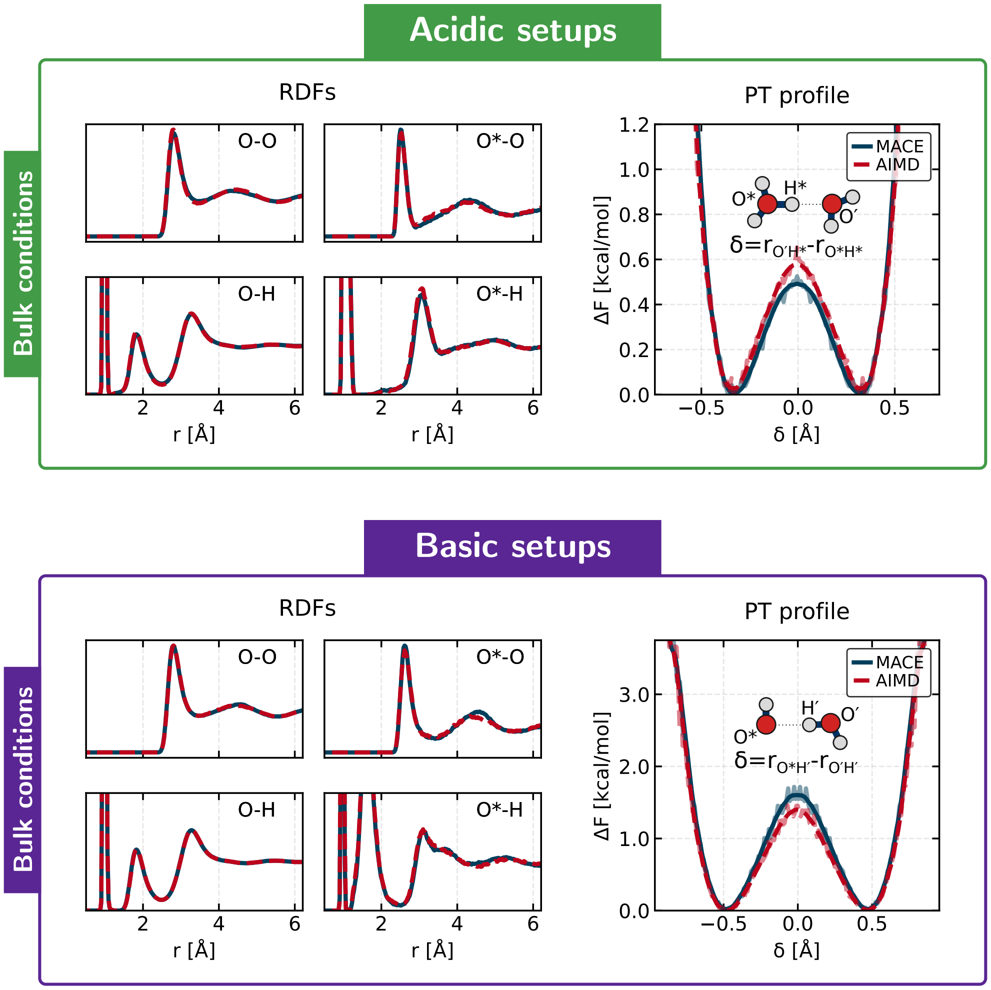

Figure S8: Comparison of RDFs and free energy profiles for protonic defect transfer in bulk conditions, using the PT coordinate defined in the schematic.

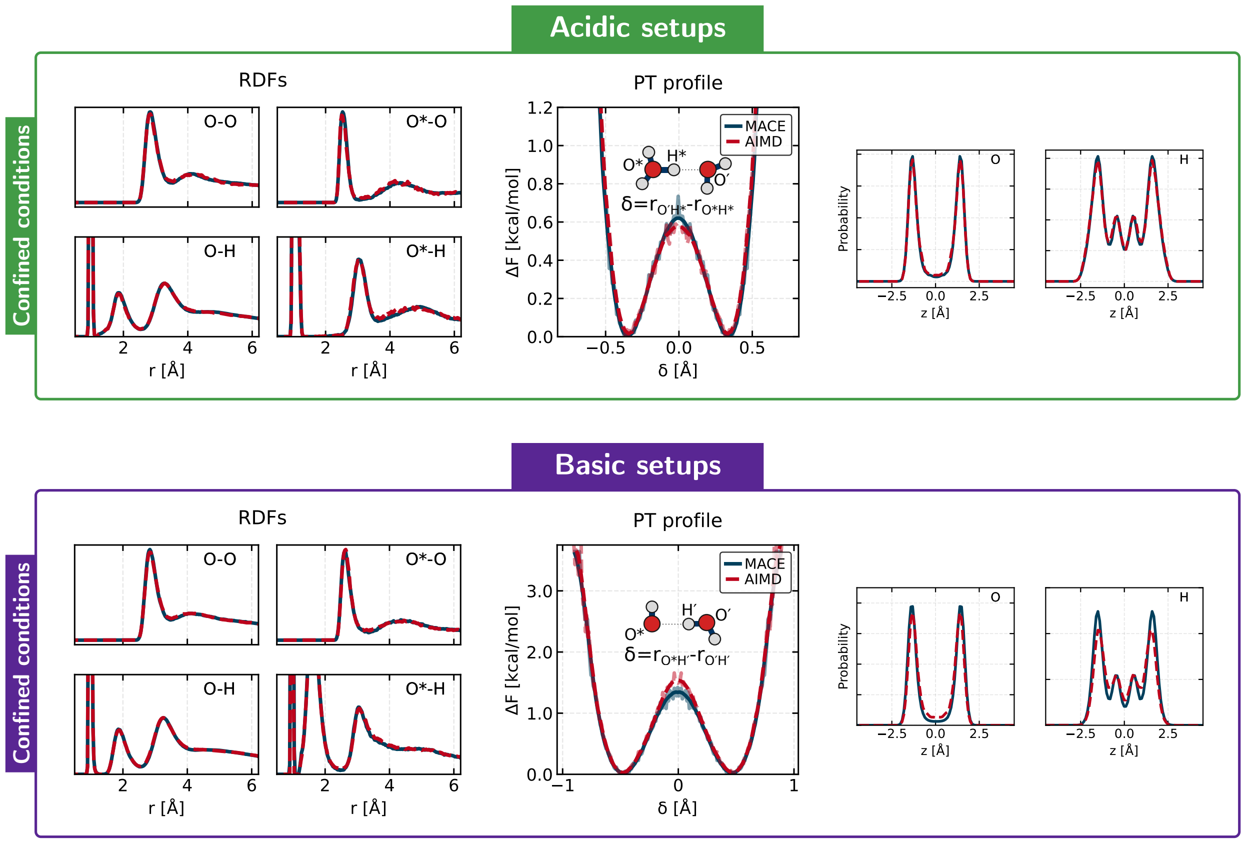

Figure S9: Comparison of RDFs, free energy profiles for protonic defect transfer, and density profiles in confined conditions, using the PT coordinate defined in the schematic.

Recovering bulk-like density

We evidence that bulk-like density is achieved at the center of the thicker slit widths by including a scale for the water density in the simulated slits presented in the main manuscript. As shown in Fig. S10, the central density closely matches that reported in Ref. S2, and serves as a benchmark for ensuring accurate densities within the slit, with the same central value referenced as 'bulk-like'.

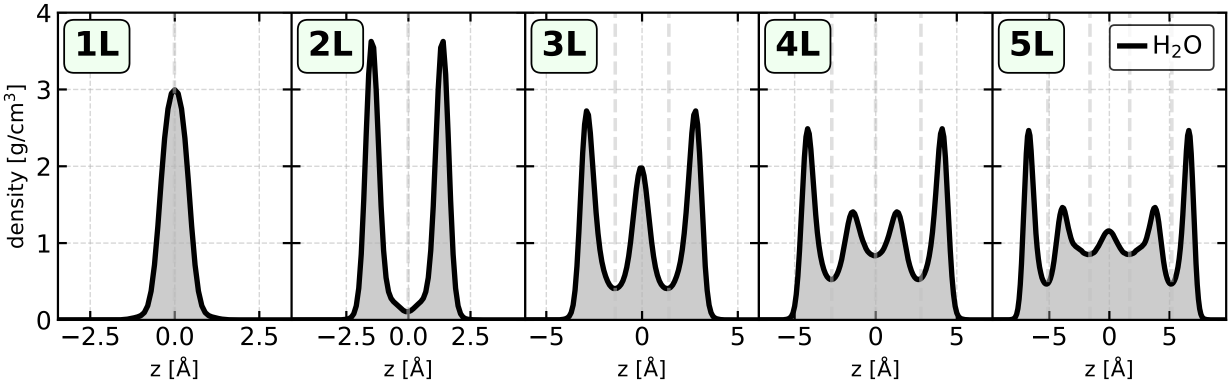

Figure S10: Water density profiles along the z axis perpendicular to the free-standing graphene sheets obtained from the oxygen atoms in a neutral water system. The vertical dashed lines indicate the partitioning among the different water layers.

Graphene-hydronium and graphene-hydroxide interactions

To further validate the graphene-hydronium and graphene-hydroxide interactions modeled with revPBE-D3, we retrained our MLP at the revPBE0-D3 level, addressing potential delocalization errors commonly associated with GGA functionals. The inclusion of exact Fock exchange in this hybrid approach allows for a more rigorous assessment of discrepancies stemming from electronic structure approximations, providing a more reliable framework for accurately capturing the interactions in question. As shown in Fig. S15, the density profiles for hydronium and hydroxide ions show near-complete overlap between revPBE-D3 and revPBE0-D3 levels, confirming that our results are robust across these electronic structure treatments. This consistency underscores the generality of our findings and suggests that the observed interactions reflect the physical behavior intrinsic to these systems, rather than artifacts of functional choice.

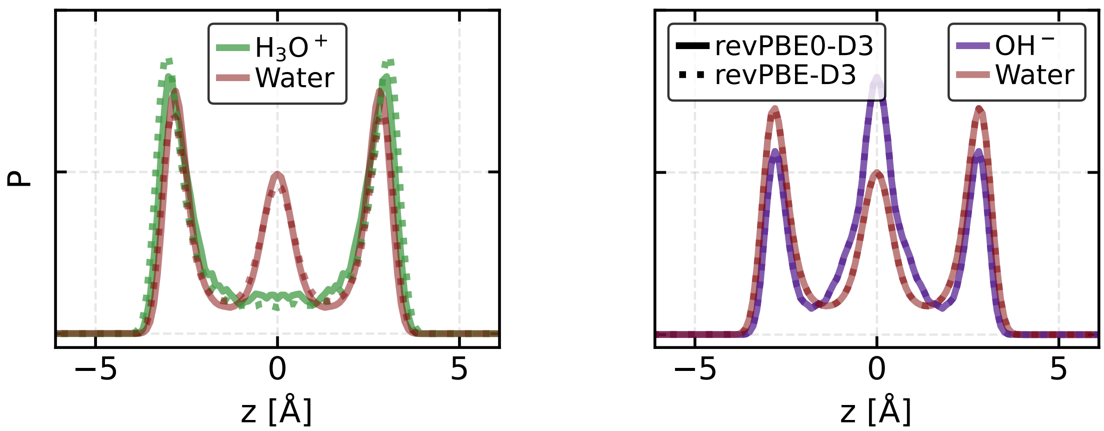

Figure S11: Normalized and symmetrized number density profiles along the z axis perpendicular to the free-standing graphene sheets obtained from the specific oxygen of the protonic defect and the oxygen of the surrounding water molecules of acidic (left) and basic (right) setups for the 3L system. The horizontal axis limits in each plot correspond to the average carbon layer positions.

We further demonstrate that the choice of dispersion correction does not significantly influence the underlying energies or forces. This is evidenced by the nearly perfect correlation observed in the parity plot comparing revPBE-D3 and revPBE-D4 energies (see Fig. S12), which shows no substantial differences between the two functionals. Furthermore, a detailed breakdown of the impact of the forces on the different atom types, including protonic defects, confirms the robustness of our approach across these corrections (see Fig. S13). This supports the reliability of our results, confirming that our conclusions remain robust regardless of the dispersion correction method used.

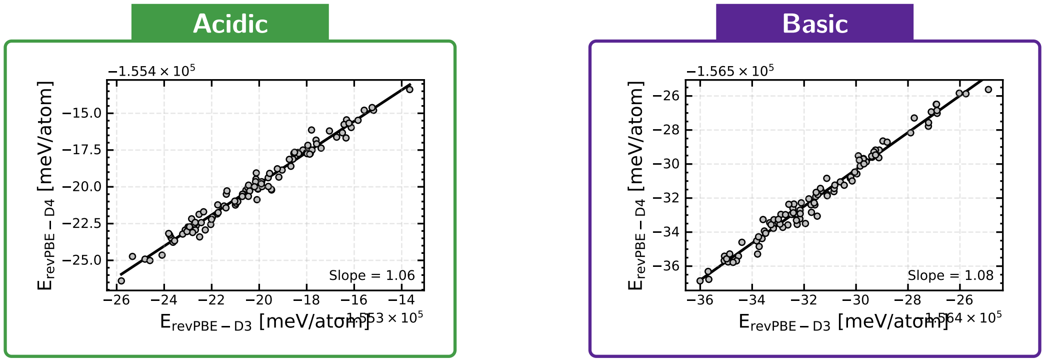

Figure S12: Parity plot for the energies using revPBE-D3 and revPBE-D4 for the acidic (left) and basic (right) 3L system.

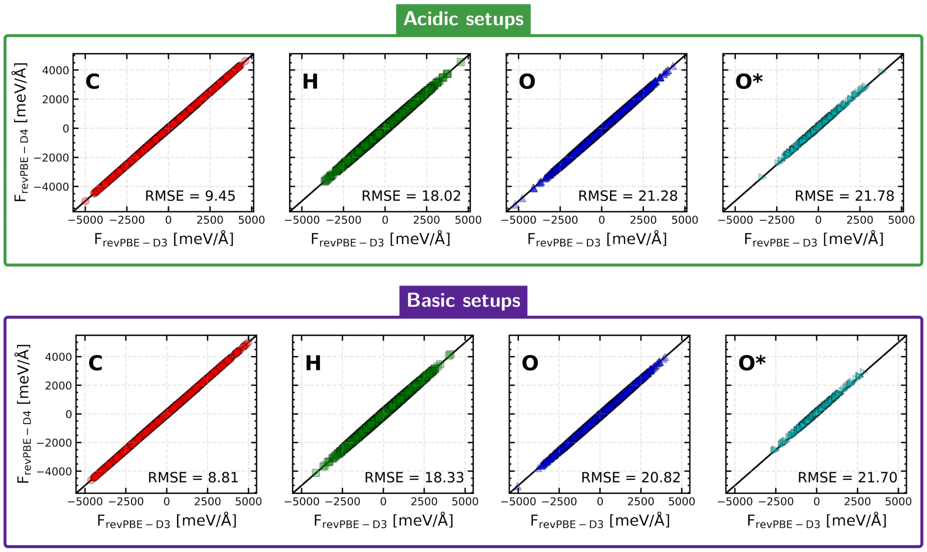

Figure S13: Parity plots for the forces obtained using revPBE-D3 and revPBE-D4, broken down by different atom types including the specific oxygen of the protonic defect O*, for the acidic (left) and basic (right) 3L system.

3 Effect of graphene flexibility

To evaluate the impact of graphene flexibility on the reported trends, we conducted additional simulations with the graphene sheets fully immobilized. To minimize computational costs, we carried out a 1 ns simulation on the smallest system, the 3L system, which features an intermediate region. This setup allowed us to efficiently observe the trends, providing a clear basis for comparison of the phenomena. Interestingly, even with the graphene immobilized, the trends in our results remained consistent. This indicates that the flexibility of the graphene sheets does not significantly affect the key trends observed in our findings, suggesting that the phenomena are robust across various mechanical constraints imposed on the graphene structure.

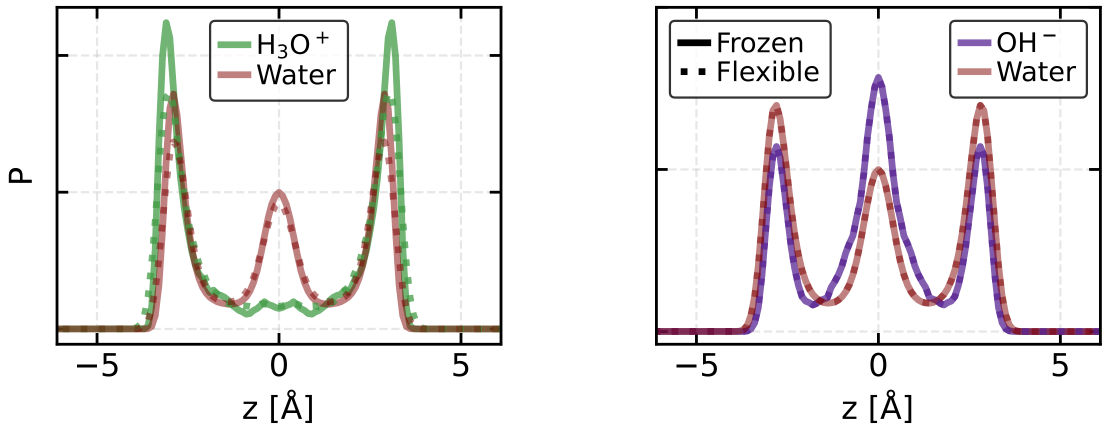

Figure S14: Normalized and symmetrized number density profiles along the z axis perpendicular to the free-standing (dashed) or frozen (solid) graphene sheets obtained from the specific oxygen of the protonic defect and the oxygen of the surrounding water molecules of acidic (left) and basic (right) setups for the 3L system. The horizontal axis limits in each plot correspond to the average carbon layer positions.

We also evaluated the influence of density (and, by extension, pressure) within these immobilized graphene slits. Our results confirm that the accumulation of protons at the graphene-water interface is insensitive to density variations within such conditions.

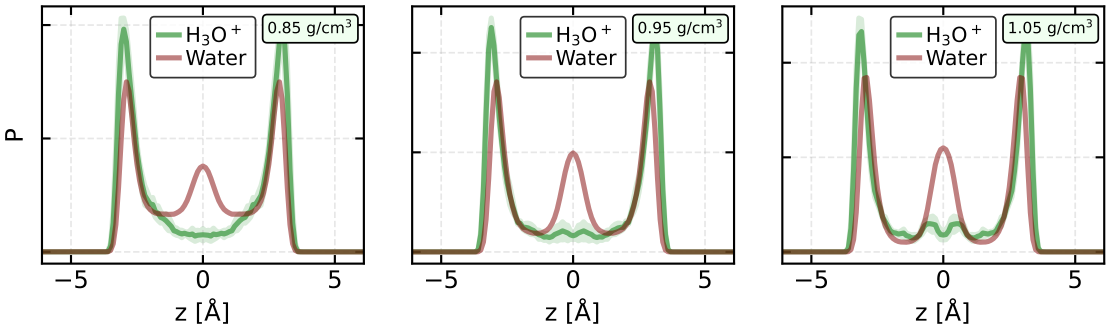

Figure S15: Normalized and symmetrized number density profiles along the z axis perpendicular to the frozen graphene sheets obtained from the specific oxygen of the protonic defect and the oxygen of the surrounding water molecules of acidic setups with varying densities (obtained using the effective volume of water) for the 3L system.

4 Potential of mean force calculation with umbrella sampling

To complement the unbiased simulations reported in the main text, we obtained the potential of mean force (PMF) of the protonic defects relative to the graphene layers using umbrella sampling.[S21,S22] For this, we conducted MD simulations where we restrained the oxygen atom O^* of the protonic defect at different target height values z_0 above an immobile (i.e., fully frozen) surface carbon atom by using a restraining potential of the form,

$$U_{\text{bias},1}(z) = \frac{k_{\text{bias},1}}{2}(z - z_0)^2 \tag{1}$$

where z is the instantaneous height of the O^* above the graphene sheet, and $k_{\text{bias},1} = 150\,\text{kcal/mol/Å}^2$. To prevent unexpected shifts in the chemical structure of protonic defects due to proton hopping –as suggested in Ref. S23, where it was applied only to the hydroxide ion– we restrained the hydrogen coordination value of the protonic defect species n_{O^*-H} around a target value n_0 using a harmonic potential of the form,

$$U_{\text{bias},2} = \frac{k_{\text{bias},2}}{2}(n_{O^*-H} - n_0)^2 \tag{2}$$

with $k_{\text{bias},2} = 400$ kcal/mol per coordination unit squared and

$$n_{O^*-H} = \sum_{i=1}^{N} \frac{1 - \left(\frac{r_i}{R_0}\right)^{12}}{1 - \left(\frac{r_i}{R_0}\right)^{20}} \tag{3}$$

where i iterates over each hydrogen atom within the simulation box (for a total of N), r_i is the distance between the hydrogen i and O^*, and R_0 is a switch distance set to 1.2 Å. In systems with a hydronium ion, the coordination number is maintained at 3.0, whereas in systems featuring a hydroxide ion, it is maintained at 1.3, corresponding to their ideal solvated

configurations.[S23] To obtain the PMF profiles, umbrella integration[S21,S22] is performed using the Python implementation from Ref. S24.

The PMF profiles obtained from umbrella integration are compared to the free energy profiles from unbiased simulations, calculated using $\Delta F = -k_{\mathrm{B}}T \ln P(z)$. This comparison, presented in Fig. S16, evidences the appropriate sampling of the phase space and the correct behavior captured in our unbiased simulations. The differences between the unbiased and biased results likely stem from the constraints introduced during the biasing process. Specifically, while the O* distance from the graphene sheet effectively captures the ion's position relative to the interface, it does not fully account for other critical degrees of freedom, such as ion orientation, which significantly influences interfacial stabilization. Additionally, constraining $n_{\mathrm{O^*-H}}$ helps maintain structural consistency but also limits proton transfer dynamics by restricting Grotthuss-like transport mechanisms.

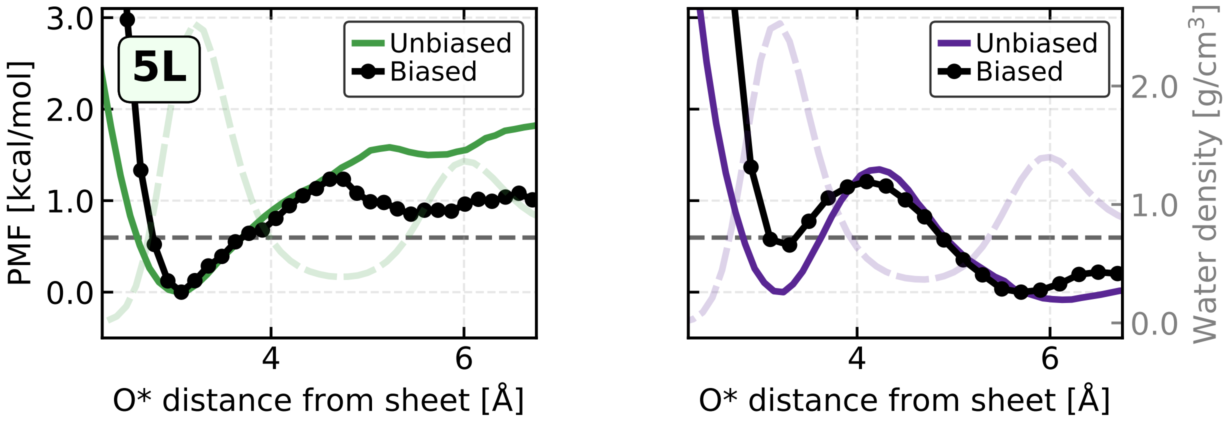

Figure S16: Potential of mean force for the hydronium (left) and hydroxide (right) ions as a function of their oxygen distance O* to the graphene sheet for the 5L system. The structuring of the water layers is represented by the water density profiles, which are indicated with corresponding lighter colors and dashed lines. The horizontal dashed line indicates the thermal energy $k_{\mathrm{B}}T \approx 0.6$ kcal/mol ($T = 300$ K).

The PMFs reported here indicate a slight destabilization of hydroxide at the interface, which is at odds with the physisorbed state reported in Ref. S23, where a free energy barrier of -0.27 ± 0.13 eV was observed for the hydroxide ion using umbrella sampling. We note in passing that no free energy barrier for the hydronium ion was reported, as this has not

yet been addressed in the literature. To provide a fair comparison of these biased PMFs and determine the root of these differences, we retrained the MLP developed herein to the PBE-D3 level to use the same settings as reported in Ref. S23. By copying their initial setup (i.e., a single layer of graphene with water on top), their simulation protocol, and their simulation times (here, we assumed a standardized interval time of 5.0 ps per umbrella window, based on their range of 4–6 ps varied according to parameter adjustments), we obtained quantitatively consistent results to those reported in their study (see Fig. S17).

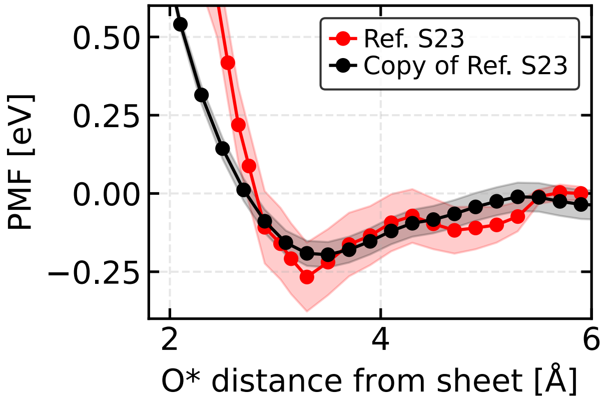

Figure S17: Potential of mean force for the hydroxide ion as a function of its oxygen distance O^* to the graphene sheet obtained from Ref. S23 (red) and that obtained using our PBE-D3 retrained MLP with the same setup (black).

However, using longer simulation times for the umbrella sampling windows (30 ps in total, including both equilibration and production time, compared to the shorter 4.5-6.5 ps), we recovered a similar free energy profile to the one reported in this manuscript, as shown in Fig. S18. This indicates that the short simulation times used in Ref. S23 led to unequilibrated structures, causing gradually increasing errors in the PMF sampling. These findings reveal the limitations of the brief simulation times employed in Ref. S23, which were constrained by the high computational cost of AIMD simulations. Notably, this also highlights the advantages of using MLPs, which have been increasingly employed in recent years to overcome the challenges associated with expensive AIMD simulations. This approach is crucial to our study as it enables us to achieve unprecedented accuracy and extensively

sample the phase space.

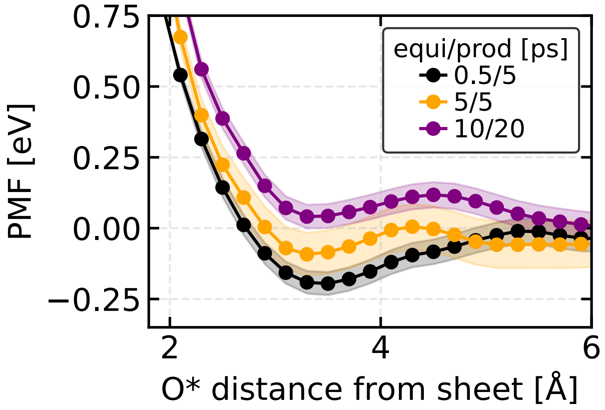

Figure S18: Potential of mean force for the hydroxide ion as a function of its oxygen distance O^* to the graphene sheet obtained by copying the setup and simulation protocol in Ref. S23 (black) and with increasingly larger equilibration/production times (yellow, purple).

5 Water hydrogen bonding

To complement the hydrogen bonding analysis of the acidic and basic systems analyzed in this work, we show in Fig. S19 the average number of hydrogen bonds donated and accepted by surrounding water molecules across different slit systems (1L to 5L). This figure demonstrates how hydrogen bonding varies with position relative to the interface.

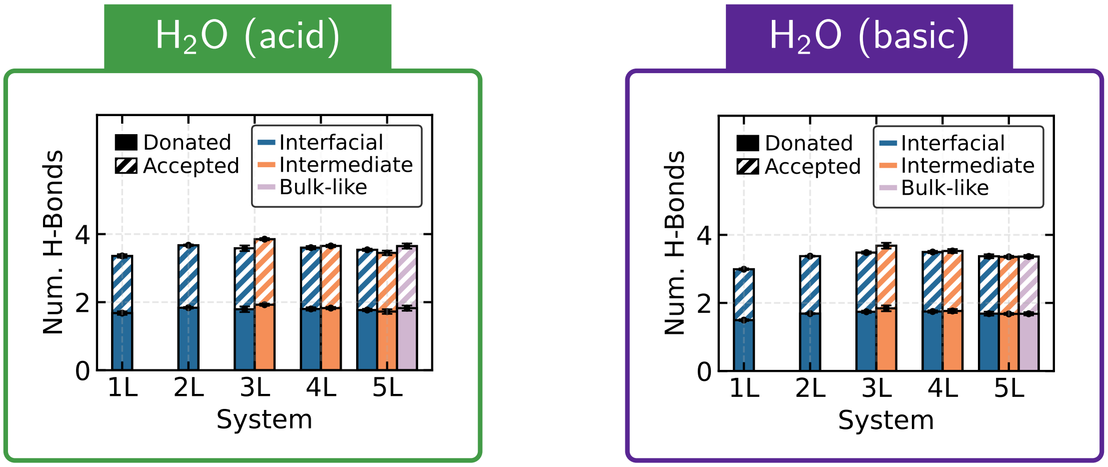

Figure S19: Average number of hydrogen bonds (accepted or donated, as indicated in each legend) for the water molecules in the acidic (left) and basic (right) systems across the different water layers. Hydrogen bonds are counted using the geometric definition provided in Ref. S25. The error bars are obtained from the standard deviation of the five replicate simulations.

6 Partial charge analysis

To determine the extent to which graphene polarizes in response to nearby hydronium or hydroxide ions, we assessed partial charges using Bader charge analysis[S26–S29] as a function of the in-plane distances from a hydronium ion or a hydroxide ion. Due to the high computational cost associated with electronic structure calculations on large systems, we analyzed the system where ions are always in close contact with the interface to ensure that polarization can be easily represented, namely the 1L system. Moreover, to further reduce the computational cost associated with this calculation, a 1050 Ry cutoff was used. While the MLP model does not explicitly include charges on carbon atoms, it does capture graphene polarizability implicitly through the DFT calculations that serve as its training data. Therefore, we utilized the MLP to generate a total of 5,000 structures sampled across the 4 ns trajectories from the MLP-based MD simulations.

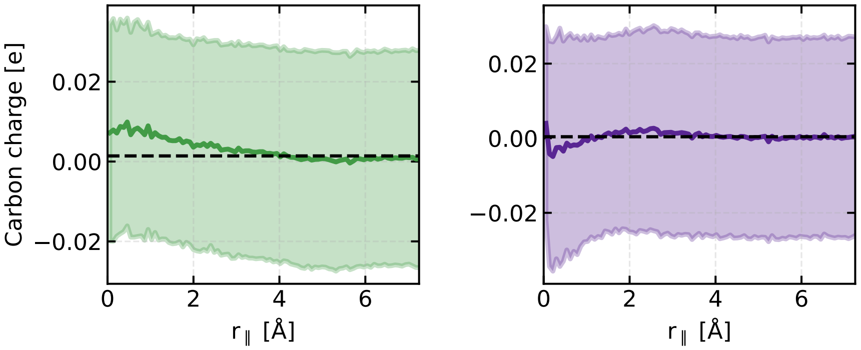

Figure S20: Carbon charges as a function of the in-plane distances from a hydronium ion (left) or a hydroxide ion (right) to the carbon atoms in the 1L system, averaged across 5,000 configurations generated by the MLP model. The shaded areas show the range of one standard deviation in the charge distribution, and the black dashed lines indicate the average carbon charge across all configurations.

It is worth noting that water molecules with an OH bond oriented toward the graphene surface can induce polarization effects similar to those of hydroxide ions. However, our analysis reveals a key difference: hydroxide ions consistently orient their OH group toward the graphene interface, creating a more stable and sustained polarization effect compared to the transient orientations observed for regular water molecules. This persistent alignment

of hydroxide at the interface underscores its distinctive interaction pattern, as clearly illustrated in Fig. S21. To further enrich this analysis, we also present the orientational profiles for hydronium ions, offering a comparative perspective on their behavior at the graphene interface.

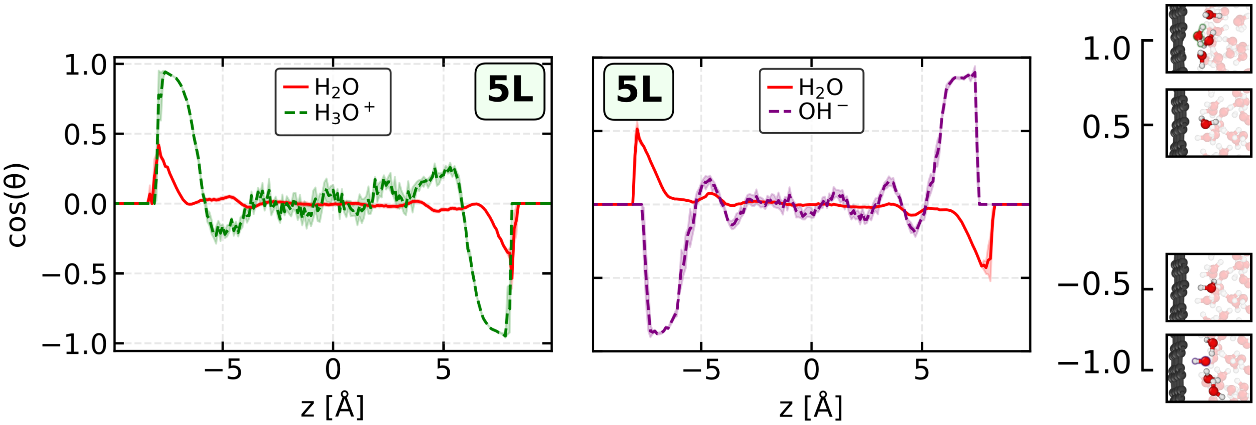

Figure S21: Average angle of the dipole of water molecules and OH^- (left) and H_3O^+ (right) along the z axis perpendicular to the free-standing graphene sheets for the 5L system with representative snapshots.

References

(S1) Castro Neto, A. H.; Guinea, F.; Peres, N. M. R.; Novoselov, K. S.; Geim, A. K. The electronic properties of graphene. *Rev. Mod. Phys.* **2009**, *81*, 109–162.

(S2) Ruiz-Barragan, S.; Muñoz-Santiburcio, D.; Marx, D. Nanoconfined Water within Graphene Slit Pores Adopts Distinct Confinement-Dependent Regimes. *The Journal of Physical Chemistry Letters* **2019**, *10*, 329–334.

(S3) Fong, K. D.; Sumić, B.; O'Neill, N.; Schran, C.; Grey, C. P.; Michaelides, A. The Interplay of Solvation and Polarization Effects on Ion Pairing in Nanoconfined Electrolytes. *Nano Letters* **2024**, *24*, 5024–5030.

(S4) Radha, B.; Esfandiar, A.; Wang, F. C.; Rooney, A. P.; Gopinadhan, K.; Keerthi, A.; Mishchenko, A.; Janardanan, A.; Blake, P.; Fumagalli, L.; Lozada-Hidalgo, M.; Garaj, S.; Haigh, S. J.; Grigorieva, I. V.; Wu, H. A.; Geim, A. K. Molecular transport through capillaries made with atomic-scale precision. *Nature* **2016**, *538*, 222–225.

(S5) Wang, Y.; Tang, F.; Yu, X.; Ohto, T.; Nagata, Y.; Bonn, M. Heterodyne-Detected Sum-Frequency Generation Vibrational Spectroscopy Reveals Aqueous Molecular Structure at the Suspended Graphene/Water Interface. *Angewandte Chemie International Edition* **2024**, *n/a*, e202319503.

(S6) Bankura, A.; Chandra, A. Proton transfer through hydrogen bonds in two-dimensional water layers: A theoretical study based on ab initio and quantum-classical simulations. *The Journal of Chemical Physics* **2015**, *142*, 044701.

(S7) Kühne, T. D.; Iannuzzi, M.; Ben, M. D.; Rybkin, V. V.; Seewald, P.; Stein, F.; Laino, T.; Khaliullin, R. Z.; Schütt, O.; Schiffmann, F.; Golze, D.; Wilhelm, J.; Chulkov, S.; Bani-Hashemian, M. H.; Weber, V.; Borštnik, U.; Taillefumier, M.; Jakobovits, A. S.; Lazzaro, A.; Pabst, H.; Müller, T.; Schade, R.; Guidon, M.; Andermatt, S.; Holmberg, N.; Schenter, G. K.; Hehn, A.; Bussy, A.; Belleflamme, F.;

Tabacchi, G.; Glöß, A.; Lass, M.; Bethune, I.; Mundy, C. J.; Plessl, C.; Watkins, M.; VandeVondele, J.; Krack, M.; Hutter, J. CP2K: An electronic structure and molecular dynamics software package - Quickstep: Efficient and accurate electronic structure calculations. *The Journal of Chemical Physics* **2020**, *152*, 194103.

(S8) Bussi, G.; Donadio, D.; Parrinello, M. Canonical sampling through velocity rescaling. *The Journal of Chemical Physics* **2007**, *126*, 014101.

(S9) Jones, A.; Leimkuhler, B. Adaptive stochastic methods for sampling driven molecular systems. *The Journal of Chemical Physics* **2011**, *135*, 084125.

(S10) Perdew, J. P.; Burke, K.; Ernzerhof, M. Generalized Gradient Approximation Made Simple. *Physical Review Letters* **1996**, *77*, 3865–3868.

(S11) Grimme, S.; Antony, J.; Ehrlich, S.; Krieg, H. A consistent and accurate ab initio parametrization of density functional dispersion correction (DFT-D) for the 94 elements H-Pu. *The Journal of Chemical Physics* **2010**, *132*, 154104.

(S12) Goedecker, S.; Teter, M.; Hutter, J. Separable dual-space Gaussian pseudopotentials. *Physical Review B* **1996**, *54*, 1703–1710.

(S13) VandeVondele, J.; Hutter, J. Gaussian basis sets for accurate calculations on molecular systems in gas and condensed phases. *The Journal of Chemical Physics* **2007**, *127*, 114105.

(S14) Larsen, A. H.; Mortensen, J. J.; Blomqvist, J.; Castelli, I. E.; Christensen, R.; Dułak, M.; Friis, J.; Groves, M. N.; Hammer, B.; Hargus, C.; Hermes, E. D.; Jennings, P. C.; Jensen, P. B.; Kermode, J.; Kitchin, J. R.; Kolsbjerg, E. L.; Kubal, J.; Kaasbjerg, K.; Lysgaard, S.; Maronsson, J. B.; Maxson, T.; Olsen, T.; Pastewka, L.; Peterson, A.; Rostgaard, C.; Schiøtz, J.; Schütt, O.; Strange, M.; Thygesen, K. S.; Vegge, T.; Vilhelmsen, L.; Walter, M.; Zeng, Z.; Jacobsen, K. W. The atomic sim-

ulation environment—a Python library for working with atoms. *Journal of Physics: Condensed Matter* **2017**, *29*, 273002.

(S15) Thompson, A. P.; Aktulga, H. M.; Berger, R.; Bolintineanu, D. S.; Brown, W. M.; Crozier, P. S.; in 't Veld, P. J.; Kohlmeyer, A.; Moore, S. G.; Nguyen, T. D.; Shan, R.; Stevens, M. J.; Tranchida, J.; Trott, C.; Plimpton, S. J. LAMMPS - a flexible simulation tool for particle-based materials modeling at the atomic, meso, and continuum scales. *Computer Physics Communications* **2022**, *271*, 108171.

(S16) Tribello, G. A.; Bonomi, M.; Branduardi, D.; Camilloni, C.; Bussi, G. PLUMED 2: New feathers for an old bird. *Computer Physics Communications* **2014**, *185*, 604–613.

(S17) Ravindra, P.; Advincula, X. R.; Schran, C.; Michaelides, A.; Kapil, V. Quasi-one-dimensional hydrogen bonding in nanoconfined ice. *Nature Communications* **2024**, *15*, 7301.

(S18) Schran, C.; Brezina, K.; Marsalek, O. Committee neural network potentials control generalization errors and enable active learning. *The Journal of Chemical Physics* **2020**, *153*, 104105.

(S19) Schran, C.; Thiemann, F. L.; Rowe, P.; Müller, E. A.; Marsalek, O.; Michaelides, A. Machine learning potentials for complex aqueous systems made simple. *Proceedings of the National Academy of Sciences* **2021**, *118*, e2110077118.

(S20) Atsango, A. O.; Morawietz, T.; Marsalek, O.; Markland, T. E. Developing machine-learned potentials to simultaneously capture the dynamics of excess protons and hydroxide ions in classical and path integral simulations. *The Journal of Chemical Physics* **2023**, *159*, 074101.

(S21) Kästner, J.; Thiel, W. Bridging the gap between thermodynamic integration and umbrella sampling provides a novel analysis method: "Umbrella integration". *The Journal of Chemical Physics* **2005**, *123*, 144104.

(S22) Kästner, J.; Thiel, W. Analysis of the statistical error in umbrella sampling simulations by umbrella integration. *The Journal of Chemical Physics* **2006**, *124*, 234106.

(S23) Grosjean, B.; Bocquet, M.-L.; Vuilleumier, R. Versatile electrification of two-dimensional nanomaterials in water. *Nature Communications* **2019**, *10*, 1656.

(S24) Stroet, M.; Deplazes, E. Umbrella integration. `https://github.com/ATB-UQ/umbrella_integration`, 2016.

(S25) Luzar, A.; Chandler, D. Hydrogen-bond kinetics in liquid water. *Nature* **1996**, *379*, 55–57.

(S26) Henkelman, G.; Arnaldsson, A.; Jónsson, H. A fast and robust algorithm for Bader decomposition of charge density. *Computational Materials Science* **2006**, *36*, 354–360.

(S27) Sanville, E.; Kenny, S. D.; Smith, R.; Henkelman, G. Improved grid-based algorithm for Bader charge allocation. *Journal of Computational Chemistry* **2007**, *28*, 899–908.

(S28) Tang, W.; Sanville, E.; Henkelman, G. A grid-based Bader analysis algorithm without lattice bias. *Journal of Physics: Condensed Matter* **2009**, *21*, 84204.

(S29) Yu, M.; Trinkle, D. R. Accurate and efficient algorithm for Bader charge integration. *The Journal of Chemical Physics* **2011**, *134*, 064111.
